# Comparative effects of human-equivalent low, moderate, and high dose oral prednisone intake on autoimmunity and glucocorticoid-related toxicity in a murine model of environmental-triggered lupus

**DOI:** 10.3389/fimmu.2022.972108

**Published:** 2022-10-19

**Authors:** Lauren K. Heine, Abby D. Benninghoff, Elizabeth A. Ross, Lichchavi D. Rajasinghe, James G. Wagner, Ryan P. Lewandowski, Alexa L. Richardson, Quan-Zhen Li, John P. Buchweitz, Justin Zyskowski, Ashleigh N. Tindle, Anna E. Skedel, Nicholas J. Chargo, Laura R. McCabe, Jack R. Harkema, James J. Pestka

**Affiliations:** ^1^ Department of Pharmacology and Toxicology, Michigan State University, East Lansing, MI, United States; ^2^ Institute for Integrative Toxicology, Michigan State University, East Lansing, MI, United States; ^3^ Department of Animal, Dairy and Veterinary Sciences, School of Veterinary Medicine, Utah State University, Logan, UT, United States; ^4^ Department of Food Science and Human Nutrition, Michigan State University, East Lansing, MI, United States; ^5^ Department of Pathobiology and Diagnostic Investigation, Michigan State University, East Lansing, MI, United States; ^6^ Department of Immunology and Internal Medicine, Institute fo Innovations in Medical Technology (IIMT) Microarray Core Facility, University of Texas Southwestern Medical Center, Dallas, TX, United States; ^7^ Toxicology Section, Michigan State University Veterinary Diagnostic Laboratory, Lansing, MI, United States; ^8^ Department of Physiology, Michigan State University, East Lansing, MI, United States; ^9^ Department of Microbiology and Molecular Genetics, Michigan State University, East Lansing, MI, United States

**Keywords:** systemic lupus erythematosus, crystalline silica, autoantibody, ectopic lymphoid tissue, lung, kidney, muscle wasting (atrophy)

## Abstract

Autoimmune diseases can be triggered by environmental toxicants such as crystalline silica dust (cSiO_2_). Here, we characterized the dose-dependent immunomodulation and toxicity of the glucocorticoid (GC) prednisone in a preclinical model that emulates onset and progression of cSiO_2_-triggered lupus. Two cohorts of 6-wk-old female NZBWF1 mice were fed either control AIN-93G diet or one of three AIN-93G diets containing prednisone at 5, 15, or 50 mg/kg diet which span human equivalent oral doses (HED) currently considered to be low (PL; 5 mg/d HED), moderate (PM; 14 mg/d HED), or high (PH; 46 mg/d HED), respectively. At 8 wk of age, mice were intranasally instilled with either saline vehicle or 1 mg cSiO_2_ once weekly for 4 wk. The experimental plan was to 1) terminate one cohort of mice (n=8/group) 14 wk after the last cSiO_2_ instillation for pathology and autoimmunity assessment and 2) to maintain a second cohort (n=9/group) to monitor glomerulonephritis development and survival. Mean blood concentrations of prednisone’s principal active metabolite, prednisolone, in mice fed PL, PM, and PH diets were 27, 105, 151 ng/ml, respectively, which are consistent with levels observed in human blood ≤ 12 h after single bolus treatments with equivalent prednisone doses. Results from the first cohort revealed that consumption of PM, but not PL diet, significantly reduced cSiO_2_-induced pulmonary ectopic lymphoid structure formation, nuclear-specific AAb production, inflammation/autoimmune gene expression in the lung and kidney, splenomegaly, and glomerulonephritis in the kidney. Relative to GC-associated toxicity, PM diet, but not PL diet, elicited muscle wasting, but these diets did not affect bone density or cause glucosuria. Importantly, neither PM nor PL diet improved latency of cSiO_2_-accelerated death. PH-fed mice in both cohorts displayed robust GC-associated toxicity including body weight loss, reduced muscle mass, and extensive glucosuria 7 wk after the final cSiO_2_ instillation requiring their early removal from the study. Taken together, our results demonstrate that while moderate doses of prednisone can reduce important pathological endpoints of cSiO_2_-induced autoimmunity in lupus-prone mice, such as upstream ectopic lymphoid structure formation, these ameliorative effects come with unwanted GC toxicity, and, crucially, none of these three doses extended survival time.

## Introduction

Systemic lupus erythematosus (lupus) is a devastating chronic human autoimmune disease that primarily affects women of childbearing age. Lupus affects multiple organs and is associated with unresolved inflammation and inadequate clearance of dying cells that precipitates loss of immunological tolerance and production of autoantibodies (AAb). These AAb form immune complexes with autoantigens (AAg) that deposit in tissues provoking recruitment of inflammatory cells, cytokine release, complement activation, and cell death in multiple organ systems (1–3). Lupus typically involves recurrent, intermittent increases in disease activity known as flares that collectively promote irreversible organ damage often manifesting in glomerulonephritis and end-stage renal disease. While genetic predisposition is a major contributor to an individual’s propensity to develop lupus, environmental triggers such as toxicants, UV radiation, infections, or drugs can play critical roles in disease onset, flaring, and progression (2, 4–7).

Exposure to respirable crystalline silica (cSiO_2_) dust particles in occupations such as construction, mining, and stonecutting is etiologically associated with the occurrence of lupus and other autoimmune diseases (6, 8–11). Consistent with epidemiologic investigations, our laboratory has developed a novel preclinical model that emulates gene-environment interaction in cSiO_2_-triggered lupus. This model employs lupus-prone female NZBWF1 mice which express key lupus hallmarks including unresolved inflammation, loss of immunological tolerance, AAb production, and glomerulonephritis that are not evident in wild-type mice without lupus predilection (12–14). Repeated exposure of female NZBWF1 mice to cSiO_2_ leads to accelerated AAb production and immune complex-mediated glomerulonephritis approximately two to three months earlier (~24 weeks of age) compared to vehicle-treated counterparts (~32-34 weeks of age).

At the mechanistic level, pulmonary exposure of NZBWF1 mice to cSiO_2_ induces production of proinflammatory cytokines and chemokines, Type 1 interferon-regulated gene expression, immunogenic macrophage death, and AAg release that together promote early loss of immunological tolerance. This loss is heralded by development of pulmonary ectopic lymphoid structures (ELS) containing follicular dendritic, T-, and B-cells that foster development of germinal centers and AAb-producing plasma cells (15). Resultant AAbs enter the systemic circulation, form immune complexes, and can deposit in the kidney, eliciting glomerulonephritis and early death (13, 16). We have extensively used this preclinical model to demonstrate that dietary intervention with the omega-3 fatty acid docosahexaenoic acid (DHA) is extremely effective in ameliorating environmental-triggered lupus onset and progression (14, 16–19).

Glucocorticoids (GC) have been a mainstay treatment for autoimmune diseases since the 1950’s (20). Prolonged GC treatment can lead to many deleterious side-effects such as muscle and bone loss, diabetes, cardiovascular disease, and increased risk of secondary infection (20–24). Patients with lupus often rely on GCs for the remainder of their lives, putting them at greater risk for GC-induced toxicity (25). The GC prednisone is often used to treat lupus flares and maintain remission due to its potent anti-inflammatory properties and because it is cost-effective compared to other treatments such as biologics (26). Prednisone doses for treatment of human rheumatological diseases have been classified as low (≤7.5 mg/d), moderate (>7.5 mg ≤30 mg/d), and high (>30 mg ≤ 100 mg/d) (27). Typical prednisone dosing regimens for patients with lupus range between 10 to 30 mg/d for treating active flaring and 5 mg/day for maintaining clinically quiescent disease (28). Chronic clinical usage of prednisone doses ranging from 6 to 7.5 mg/day are suggested thresholds for minimizing adverse effects and organ damage (29–32).

Much of what is known about prednisone in lupus treatment is derived from clinical investigations evaluating i) its effectiveness in alleviating disease flaring and reducing organ damage and ii) its capacity for eliciting untoward side effects. However, there is a surprising lack of preclinical studies elucidating underlying mechanisms for quelling lupus flaring and progression or identifying toxic thresholds for this potent drug. Moreover, little is known about the formation of pulmonary ELS in the context of lupus and how GCs can regulate ELS when they act as a nexus for autoimmunity. The purpose of the present investigation was to test the hypotheses that oral administration prednisone *via* the diet dose-dependently 1) suppresses cSiO_2_-induced inflammation and autoimmunity and 2) induces secondary GC-induced toxicity in female NZBWF1 mice.

## Materials and methods

### Animals and diets

**Figure 1:**
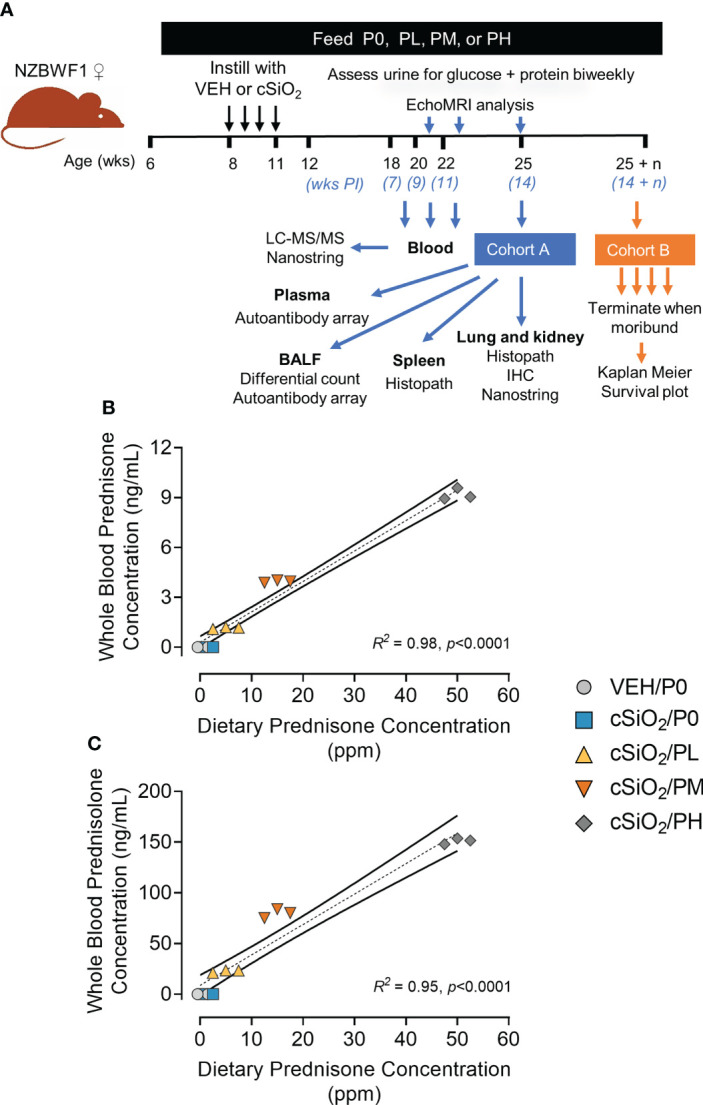
Experimental design and confirmation of prednisone and prednisolone levels in whole blood. **(A)** Female NZBWF1 mice obtained at 6 wk of age were immediately placed on either control AIN-93G diet containing no prednisone (P0) or prednisone-amended diets. Diets were maintained throughout the duration of the study. Beginning at 8 wk of age, mice were intranasally instilled with either saline vehicle (VEH) or 1 mg of cSiO_2_ once per week for 4 consecutive weeks. Eight animals per treatment group (Cohort A) were sacrificed at 25 wk of age to evaluate relevant endpoints for onset of pulmonary and renal autoimmunity. The remaining 9 mice per treatment group (Cohort B) were maintained until they met moribund criteria and were humanely sacrificed. Health and proteinuria were monitored following cSiO_2_ instillations to evaluate disease progression. Food intake, body weights, and fat/lean muscle mass composition were monitored as well to assess prednisone-induced toxicity. Levels of prednisone **(B)** and its active metabolite, prednisolone **(C)** in whole blood samples taken from mice at 19 wk of age were determined by LC-MS/MS. Samples were pooled within each cage (n=3/cage), with 3 cages per treatment group resulting in an n=3/group for analysis. Prednisone concentration detected in whole blood is predictable based on dietary prednisone concentrations. For regression analyses, R^2^ and p-values are reported. Shaded bands around regression lines represents 95% confidence intervals.

Experimental animal procedures were approved by the MSU Institutional Animal Care and Use Committee (AUF #PROTO201800113) in accordance with the guidelines of the National Institute of Health. Female 6-week-old NZBWF1 mice were obtained from Jackson Laboratories (Bar Harbor, ME), and upon arrival were randomly divided into two cohorts, Cohort A (n=40, 8 per group) and Cohort B (n=45, 9 per group), housed 4 per cage or 3 per cage, respectively (**Figure 1A**). Mice were kept under a 12-hr light/dark cycle with free access to both food and water at constant temperature and humidity (21°C-24°C and 40-55%, respectively).

Purified, powdered American Institute of Nutrition (AIN)-93G diet containing 70 g/kg oil was prepared as previously described (19). All diets contained 60 g/kg food-grade high-oleic safflower oil and 10 g/kg food-grade corn oil to supplement essential fatty acids (**Supplementary Table 1**). Unamended AIN-93G was used as control (P0) diet. For treatment diets, 500 mg prednisone (USP-grade, anhydrous, micronized, Spectrum Chemical) was first dissolved in a minimal warm ethanol and then added to 250 g of CON diet. After mechanical mixing and drying overnight in the chemical fume hood, the concentrate was mixed with an additional 750 g CON diet to yield a concentrated prednisone-amended diet stock of 500 mg/kg. This concentrated working stock was then mixed thoroughly with CON AIN-93G using a Kitchen Aid Mixer to achieve low (PL, 5 mg/kg diet), medium (PM, 15 mg/kg diet), and high (PH, 50 mg/kg diet) concentrations (**Table 1**). These diet concentrations are estimated to achieve human equivalent doses (HED) of 5, 14, and 46 mg/d, respectively, using an average body weight of 77 kg for women ≥ 20 years of age in the U.S (32). Prednisone content was verified in representative experimental diets by LC-MS/MS. Diets were prepared bi-weekly and stored at -20°C and provided *ad libitum* in feed jars designed to minimize spillage during the study. Diet intake for Cohort A mice was measured each week to determine dietary intake/mouse/24 h to determine if prednisone caused hyperphagia or hypophagia (**Supplementary Figure 1**) (35). These measurements indicated each mouse consistently consumed approximately 3-4 grams of diet per 24 h.

**Table 1:** Relation of prednisone concentrations in experimental diet to mouse and human daily doses.

Experimental diet^a^	Prednisone			
	Concentration^b^ (mg/kg diet)	Mouse dose^c^ (mg/kg bw/d)	HED^d^ (mg/kg bw/d)	HED^e^ (mg/d)
P0 (CON)	0 (0)	0	0	0
PL	5 (5.0)	0.75	0.06	5
PM	15 (15.2)	2.25	0.18	14
PH	50 (49.8)	7.5	0.6	46

a P0(CON) refers to control diet; PL, PM and PH correspond to low, moderate and high dose prednisone diets.

b Values in parentheses refer to LC-MS analysis of a representative lot of experimental diets.

c Mouse dose was calculated by multiplying dietary concentration by the FDA conversion factor of 0.15 (33).

d Human equivalent dose was-calculated by multiplying mouse dose by FDA drug interspecies conversion factor of 0.08 (34).

e Daily human equivalent dose based on average body weight of 77 kg for women ≥ 20 years of age in the U.S (32).

### Experimental design

**Figure 1A** depicts experimental design employed for this study. Briefly, 6 wk old NZBWF1 mice were acclimated to P0 diet for 3 days, then groups from Cohorts A and B were administered P0, PL, PM, or PH diet for the duration of the study. Two weeks after initiating experimental diets, mice were anesthetized with 4% isoflurane and intranasally instilled with either with 1.0 mg cSiO_2_ (Min-U-Sil-5, 1.5-2.0 µm average particle size, Pennsylvania Sand Glass Corporation, Pittsburgh, PA) in 25 µl sterile phosphate buffered saline (PBS) or with 25 µL PBS vehicle (VEH) once per wk for 4 wk as described previously (13). Starting at 8 wk post final instillation (PI), urine was collected bi-weekly and evaluated for proteinuria (defined as ≥ 300 mg/dL) and glucose using reagent dipsticks (Cortez Diagnostics, Calabasas, CA). Blood was collected *via* saphenous vein on all Cohort B mice at 7, 9, and 11 wk PI between 8:30 and 11:30 AM for prednisone and prednisolone analysis. The initial plan was for all Cohort A mice were to be sacrificed at 14 wk PI, while Cohort B mice were to be maintained until they displayed moribund criteria (**Supplementary Table 2**). However, PH-fed Groups A and B mice exhibited extensive glucosuria at 7 wk PI requiring their early termination.

### Animal necropsy and tissue selection

Mice were euthanized *via* intraperitoneal injection with sodium pentobarbital at 56 mg/kg body weight. Using heparinized syringes, blood was collected from the abdominal aorta and centrifuged at 3500 x g for 10 min at 4°C to separate the erythrocytes from plasma, and then stored at -80°C. Plasma was later analyzed for blood urea nitrogen (BUN) levels using a colorimetric detection kit (Thermo Fisher Scientific, Wilmington, DE). Bronchoalveolar lavage fluid (BALF) was collected from the lung as described previously (36). The left lung lobe was fixed in 10% neutral buffered formalin (NBF) (Fisher Scientific, Pittsburgh, PA) at a constant pressure of 30 cm H_2_O for a minimum of 1 h, then immersed in a large volume of the fixative until further routine processing for light microscopic examination and morphometric analyses described below. Spleens were weighed, then half was stored in NBF along with one kidney until processed for histology. The caudal lung lobe and half a kidney were stored individually in RNAlater (Thermo Fisher Scientific, Wilmington, DE) at 4°C. The remainder of the right lung, half kidney, and half spleen were snap-frozen in liquid nitrogen and stored at -80°C.

### Prednisone and prednisolone analyses

Experimental diets were analyzed to verify prednisone concentrations and whole blood was evaluated for prednisone and its active metabolite prednisolone by LC-MS/MS at the MSU Veterinary Diagnostic Laboratory as described in Supplementary Methods.

### EchoMRI for muscle and fat composition

An EchoMRI™ (Echo Medical Systems LLC, Houston, TX) was used to measure live body lean muscle and fat composition at 21, 23, and 25 wk of age as described previously (37). Mice were weighed just prior to live body measurement. Random errors in measurements were minimized by using a 4 min scan duration and thereby increasing the number of primary accumulations per body scan.

### Inflammatory cells in BALF

Total leukocytes in the collected BALF were determined using a hemacytometer. Cytological slides were prepared by centrifuging BALF at 400 x g for 10 min using the Shandon Cytospin 3 (Shandon Scientific, PA) and stained with Diff-Quick (Fisher Scientific). Differential cell counts for macrophages/monocytes, neutrophils, lymphocytes, and eosinophils in BALF were assessed using morphological criteria from a total of 200 total cells. Remaining BALF was centrifuged at 2400 x g for 15 min, and supernatant was stored at -80°C for subsequent AAb microarray.

### Tissue selection for lung histology

Randomly selected, transverse tissue blocks were selected from inflation-fixed left lung lobes. Tissues were paraffin embedded, sectioned at a thickness of 5 microns, and were stained with hematoxylin and eosin (H&E) for light microscopic examination. Tissue sections were semi-quantitatively scored for histopathology (lesion severity) by a board-certified veterinary pathologist (JRH) using the following criteria (% of total lung tissue section affected): (0) no lesions associated with exposure, (1) minimal (<10%), (2) mild (10< 25%), (3) moderate (25< 50%), (4) marked (50< 75%), (5) severe (>75%). Lungs were evaluated for the following lung lesions: (a) ectopic lymphoid tissue (ELS), (b) alveolar proteinosis, and (c) inflammation (alveolitis).

### Lung immunohistochemistry and morphometry

Formalin-fixed, paraffin-embedded lung sections were immunohistochemically stained to identify B and T lymphocytes, and plasma cells, using CD45R, CD3, and IgG antibodies (**Supplementary Table 3**), respectively, as previously described in detail (19). Slides were digitally scanned using the VS110 virtual slide system (Olympus, Hicksville, NY). Two hundred scanned images at 20x magnification were randomly sampled using systematic random sampling NewCast software (Visiopharm, Hoersholm, Denmark). Using the STEPanizer 1.8 Stereology Tool (38), pulmonary CD45R^+^, CD3^+^, and IgG^+^ lymphoid cells were counted by overlaying a point grid on randomly sampled images (38). Total cell densities were calculated by dividing the number of grid points overlayed with positively stained cells by reference tissue area to get a percentage of total lung area showing positive staining.

### Kidney histopathology

Kidney tissue sections (5 µm) were histochemically stained with periodic acid Schiff and hematoxylin for microscopic examination for renal histopathology. Sections were semi-quantitatively scored (JRH) based on a modified International Society of Nephrology/Renal Pathology Lupus Nephritis Classification system (39) as follows: (0) no tubular proteinosis, normal glomeruli; (1) mild tubular proteinosis with multifocal segmental proliferative glomerulonephritis and occasional early glomerular sclerosis and crescent formation; (2) moderate tubular proteinosis with diffuse segmental proliferative glomerulonephritis, early glomerular sclerosis, and crescent formation; (3) marked tubular proteinosis with diffuse global proliferative and sclerosing glomerulonephritis.

### Autoantibody microarray profiling

BALF and plasma IgG AAb profiling was performed using a high-throughput autoantigen (AAg) microarray at the Microarray and Immune Phenotyping Core Facility at the University of Texas Southwestern Medical Center as we described previously (18). Briefly, BALF and plasma samples were pre-treated with DNAse I to remove free-DNA, then diluted at a 1:25 or 1:50 dilution, respectively. Samples were added and hybridized to protein array plates containing 122 different antigens and 6 controls. Antibody-antigen binding occurring on the plate was detected using Cy3-conjugated anti-mouse IgG (1:2000, Jackson ImmunoResearch Laboratories, PA). Fluorescent images were captured using a Genepix 4200A scanner (Molecular Devices, CA) and were transformed to signal intensity values using GenePix 7.0 software. Signal intensity values for each AAb were first normalized by subtracting the background and normalizing to IgG internal controls, and then reported as an antibody score (Ab-score). This Ab-score was calculated based on the normalized signal intensity (NSI) and signal-to-noise ratio (SNR) using the formula:


$$Ab-score=log_{2}\;(NSI*SNR+1)$$

Normalized and unit variance-scaled Ab-score values were visualized using ClustVis software (40). Data were clustered using unsupervised hierarchical co-clustering (HCC). Rows were clustered using Euclidean distance and Ward linkage. Imputation was applied for missing value estimation. Selected AAb-scores were reported as violin plots, generated using Prism 9 (GraphPad Prism v 9.2, San Diego, CA).

### NanoString autoimmune gene profiling

RNA was extracted from lungs and kidneys of Cohort A mouse groups with RNeasy Mini Kits with DNase treatment (Qiagen, Valencia, CA). RNA was dissolved in nuclease-free water, quantified with Qubit (Thermo Fisher Scientific), and integrity verified with a TapeStation (Agilent Technologies). Samples (RNA integrity >8) were analyzed with NanoString Autoimmune Gene Expression assay (XT-CSO-MAIP1-12, NanoString Technologies, Seattle, WA) at the MSU Genomics Core. Assays were performed and quantified on the nCounter MAX system, sample preparation station, and digital analyzer (NanoString Technologies) according to the manufacturer’s instructions.

Raw gene expression data were analyzed using NanoString’s software nSolver v3.0.22 with the Advanced Analysis Module v2.0. Background subtraction was performed using the eight negative controls included with the module. Genes with counts below a threshold of 2σ of the mean background signal were excluded from subsequent analysis. Data normalization was performed on background-subtracted samples using internal positive controls and selected housekeeping genes that were identified with the geNorm algorithm (https://genorm.cmgg.be/) (**Supplementary Figure 2**).

Differential gene expression analyses were performed as previously described (41, 42) using the nSolver Advanced Analysis Module, which employs several multivariate linear regression models (mixture negative binomial, simplified negative binomial, or log-linear model) to identify significant genes (**Supplementary Figure 3**). Resulting *p* values were adjusted using the Benjamini-Hochberg (BH) method to control the false discovery rate (**Supplementary File 1**). A statistically significant difference in gene expression was defined as 1.5-fold change in expression (log_2_ >0.58 or<-0.58) with BH q<0.05. Four pairwise comparisons within each time point for each tissue examined were determined a priori, as follows: cSiO_2_/P0 vs VEH/P0, cSiO_2_/PL vs cSiO_2_/P0, cSiO_2_/PM vs cSiO_2_/P0, and cSiO_2_/PM vs cSiO_2_/PL. Expression data for lung, kidney, and blood samples are available as normalized linear counts in **Supplementary File 2**. Venn diagrams of significant differentially expressed genes were generated using BioVenn (43).

To assess the impact of experimental diets on annotated gene sets, global and directed significance scores were calculated for each pathway at each time point, as previously described (42). The global score estimates the cumulative evidence for the differential expression of genes in a pathway. Directed significance scores near zero indicate that a pathway may have many highly regulated genes, but no apparent tendency for those genes to be over- or under-expressed collectively. As a complementary method for comparing pathways and discriminating between experimental groups, pathway Z scores were calculated as the Z-scaled first principal component of the pathway genes’ normalized expression. ClustVis (40) was used to perform unsupervised hierarchical cluster analyses (HCC) and principal components analyses (PCA) using log_2_ transcript count data for DEGs. Spearman rank correlations were performed to examine overall patterns in the gene expression profiles using the pathway Z score compared to other biomarkers of disease in lung or kidney tissues at 14 weeks PI. A significant correlation was inferred when *ρ* >0.5 or <-0.5 and p<0.05. Network analyses for interactions among significant genes were performed using STRING database version 11.5 (http://string-db.org/), with a minimum interaction score > 0.05 and cluster identification using the Markov Cluster (MCL) algorithm with inflation parameter of 1.5. Networks generated by STRING were visualized with Cytoscape v. 3.9.

The NanoString nSolver Advanced Analysis software employs the method described by Danaher (44) to measure the abundance of various immune cell populations using marker genes that are expressed stably and specifically in particular cell types. Cell type scores were calculated as the average log-scale normalized expression of their characteristic genes. Relative cell type measurements were based on the total population of infiltrating lymphocytes, which is useful in a sample of heterogenous mix of cell types. Only cell types that exceeded the quality control analysis for correlation of marker gene expression are reported.

### Micro-computed tomography bone analysis

Femurs previously fixed in NBF were scanned using the GE Explore Locus micro-computed tomography system (GE Healthcare, Piscataway, NJ) as previously described (45). Analysis is based on the femoral trabecular region defined as 10% of the total bone length proximal to the distal growth plate extending towards the diaphysis, excluding cortical bone. A fixed threshold of 700 was used (determined by autothreshold and isosurface visualization tools) and all scans included both experimental groups and controls as well as a calibration phantom to normalized grayscale values. Trabecular bone volume fraction (BV/TV%) was obtained using GE Healthcare MicroView Software and was subsequently corrected for body weight (BV/TV/BW) (**Supplementary Figure 4**).

### Statistical analysis

All data were analyzed and statistical tests were performed using Prism 9 (GraphPad Prism v 9.2, San Diego, CA) except for the NanoString gene expression data discussed above. Data were assessed for outliers using the Grubb’s outlier test (with Q=1%) and for normality using the Shapiro-Wilk test (p<0.01). Data of histopathological endpoints were analyzed using an unpaired one-tailed *t*-test to detect cSiO_2_-induced inflammation and autoimmunity in lupus-prone mice (VEH/P0 vs cSiO_2_/P0) and a one-way ANOVA with Dunnett’s *post-hoc* test to address our hypothesis that dietary prednisone would dose-dependently suppress cSiO_2_-triggered responses (cSiO_2_/P0 vs cSiO_2_/PL or cSiO_2_/PM). Non-normal and semi-quantitative data were analyzed using the nonparametric Mann-Whitney U test (for VEH/P0 vs cSiO_2_/P0) and the nonparametric Kruskal-Wallis test with a Dunn’s *post-hoc* test (cSiO_2_/P0 vs cSiO_2_/PL or cSiO_2_/PM). Data are presented as mean ± standard error of the mean (SEM), with a p-value ≤ 0.05 being considered as statistically significant.

## Results

### Prednisone-amended diets dose-dependently increased blood levels of prednisone and prednisolone

LC-MS/MS revealed that consumption of the three prednisone-amended diets dose-dependently increased blood concentrations of prednisone and its active metabolite prednisolone at 19 wk of age in cSiO_2_-instilled mice (**Figures 1B, C**). Mean prednisone and prednisolone concentrations in PL-fed mice were 1.2 ± 0.03 ng/ml and 22.6 ± 0.8 ng/ml, respectively, in PM-fed mice were 4.0 ± 0.04 ng/ml prednisone and 79.8 ± 2.55 ng/ml prednisolone, respectively, and in PH-fed mice were 9.2 ± 0.2 ng/ml and 151 ± 22 ng/ml prednisolone, respectively. Mice fed CON diet had no detectable prednisone and prednisolone in the blood. Prednisone and prednisolone concentrations in the blood remained consistent at 21 and 23 weeks of age for PL- and PM-treated mice (**Supplementary Figure 5**). However, PH-treated mice were terminated at 19 wk of age because of GC-associated toxicity as described in the next section.

### Prednisone dose-dependently induced GC-associated toxicity

Mice consuming PH exhibited reduced body weight gain compared to mice fed P0 beginning at age 8 wk **(** **Supplementary Figure 6A** **)** that presaged additional GC-induced toxic effects. PH-fed mice began to lose body weight beginning at age 16 wk (4 wk PI) and displayed a significant reduction of lean muscle mass at age 17 wk (**Supplementary Figure 6B**). By age 19 wk, these mice had high levels of glucose (760 mg/dl) present in their urine indicating that they were extremely hyperglycemic (**Supplementary Figure 6C**). Due to this evidence of deteriorating health, PH-fed mice in Cohorts A and B were euthanized approximately 7 wk prior to the scheduled necropsy for Cohort A and were not included in further analyses.

**Figure 2:**
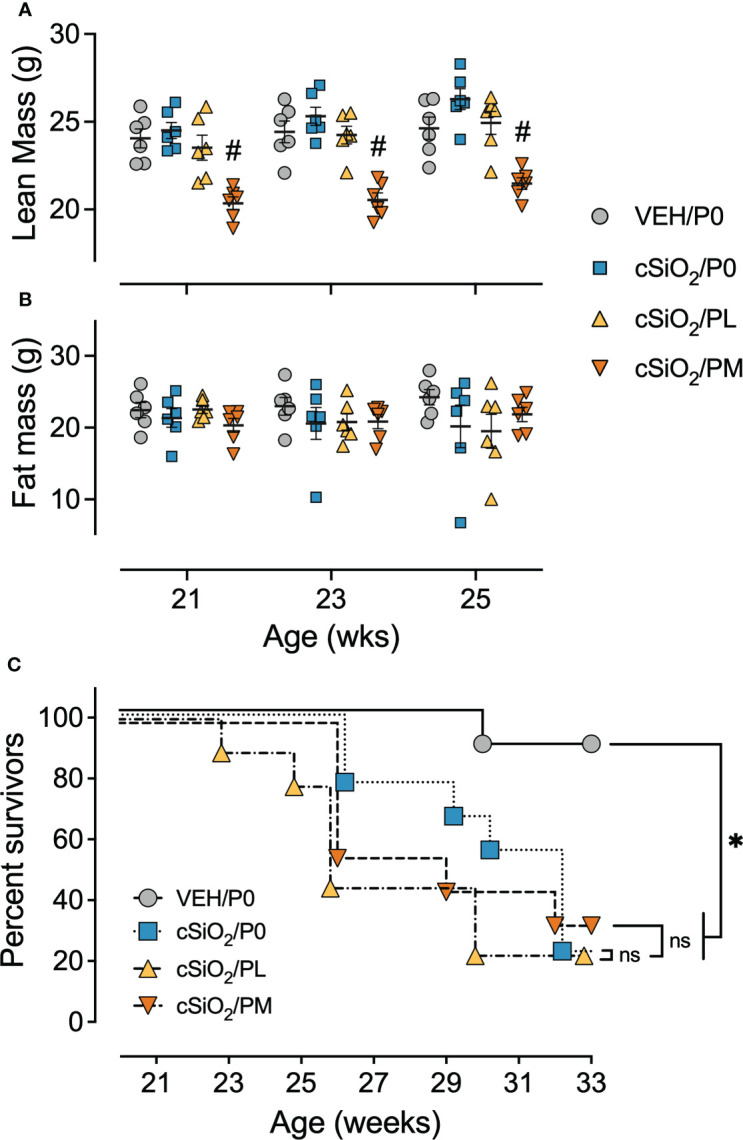
Moderate dose prednisone treatment significantly reduced lean muscle mass and did not improve survivability. Mice were evaluated for lean muscle **(A)** and fat mass **(B)** using EchoMRI at 21 wk of age (9 wk PI), 23 wk of age (11 wk PI), and 25 wk of age (13 wk PI). PM treatment induced significant lean muscle mass loss compared to cSiO_2_ control mice. cSiO_2_ and prednisone had no effects on fat mass. *Indicates p<0.05 for VEH/P0 vs cSiO_2_/P0; ^#^indicates p<0.05 for cSiO_2_/P0 vs cSiO_2_/PL or cSiO_2_/PM group as described in Materials and Methods. **(C)** Kaplan-Meier survival plot depicting survival of mice in cohort B which were evaluated weekly using moribund criteria. *Indicates p<0.05 for VEH/P0 group survival compared to cSiO_2_/P0, cSiO_2_/PL, or cSiO_2_/PM group survival as determined by the Mantel-Cox log-rank test. ns indicates not significant. No differences in survival between cSiO_2_/P0 and the cSiO_2_/PL or cSiO_2_/PM groups were observed.

Consumption of PM, but not PL, also impaired body weight gain prior to cSiO_2_ instillations starting at age 8 wk and lasting for the duration of Cohort A and B studies (**Supplementary Figure 6A**). Reduced body weights in PM-fed cSiO_2_-instilled mice corresponded with significant reductions in lean mass, but not fat mass, at age 21 wk (9 wk PI), 23 wk (11 wk PI), and 25 wk (13 wk PI), as compared to cSiO_2_/P0 mice (**Figures 2A, B** **)**. cSiO_2_ did not impact the amount of lean muscle or fat mass measured in CON- or prednisone-fed mice compared to VEH-treated CON-fed mice. Unlike muscle wasting, neither PL nor PM diet affected bone density in cSiO_2_-exposed mice (**Supplementary Figure 4**). Finally, glycosuria was not detected in VEH/P0, cSiO_2_/P0, cSiO_2_/PL, or cSiO_2_/PM groups at 19, 21, or 23 wk of age (**Supplementary Figure 6C**).

### Prednisone intake did not improve survival time in cSiO_2_-treated mice

In accordance with a previous study (16), the median survival time of Cohort B cSiO_2_/P0 mice (32 wk of age) was significantly reduced compared to VEH-treated CON-fed mice (>33 wk of age) (**Figure 2C**). PL and PM intake also had reduced median survival times of cSiO_2_-exposed mice (26 wk and 29 wk of age, respectively) suggesting that oral prednisone intake was not effective in enhancing the lifespan of cSiO_2_-exposed lupus-prone mice.

### Moderate prednisone intake suppressed cSiO_2_-induced pulmonary ELS formation

**Figure 3:**
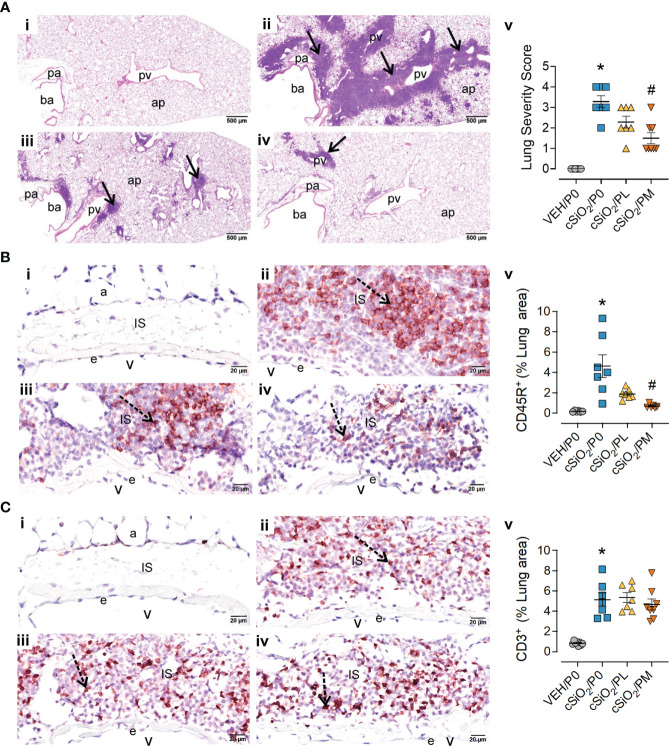
Moderate dose prednisone (PM) significantly reduced pulmonary peribronchiolar and perivascular formation of Ectopic Lymphoid Structures (ELS) and infiltration of perivascular CD45R+ B cells, but not CD3+ T cells. Light photomicrographs of hematoxylin and eosin-stained lung tissue sections **(A)** from **(i)** VEH/P0, **(ii)** cSiO_2_/P0, **(iii)** cSiO_2_/PL, and (iv) cSiO_2_/PM mice. cSiO_2_ treatment resulted in formation of ELS in peribronchiolar and perivascular interstitium (A.ii). Graphic representation of semi-quantitative severity scores following assessment criteria of (1) minimal (<10% of lung tissue affected); (2) slight (10-25%); (3) moderated (26-50%), (4) marked (51-75%), (5) severe (>75%) of total area affected (A.v). PM, but not PL, significantly reduced the amount of ELS (A.iii-v). pa, pulmonary artery; ba, bronchiolar airway; pv, pulmonary vein; alveolar parenchyma; arrows, peri-vascular/bronchiolar ectopic lymphoid structures. Photomicrographs of lung tissue immunohistochemically stained for **(B)** CD45R+ B lymphoid cells and **(C)** CD3+ T cells in lungs from **(i)** VEH/P0, **(ii)** cSiO_2_/P0, **(iii)** cSiO_2_/PL, and (iv) cSiO_2_/PM mice. e, endothelium; v, venous lumen; IS, perivascular interstitial space; a, alveolus. cSiO2 treatment significantly triggered interstitial infiltration of CD45R+ B cells (B.ii) and CD3+ T cells (C.ii). PM treatment significantly reduced CD45R+ B cell infiltration (B.iv) but had no effect on CD3+ T cells (C.iii-iv). Graphical representation of morphometrically determined density of CD45R+ B cells (B.v), and CD3+ T cells (C.v) in lung tissue. *Indicates p<0.05 for VEH/P0 vs cSiO_2_/P0; ^#^indicates p<0.05 for cSiO_2_/P0 vs cSiO_2_/PL or cSiO_2_/PM group.

Consistent with prior studies (13, 14, 16), intranasal instillation with cSiO_2_ induced conspicuous perivascular and peribronchiolar lymphoplasmacytic infiltration (ELS) compared to VEH/P0 mice (**Figures 3A.i-ii**). Semi-quantitative severity scoring of pulmonary tissues demonstrated marked ELS formation in cSiO_2_-instilled mice fed control diet (cSiO_2_/P0) compared to VEH/P0 mice (**Figure 3A.v**). Reflective of the histopathology (**Figure 3A.iv**), PM treatment significantly reduced lung severity scores. cSiO_2_ instillation elicited significant increases in inflammatory cells (neutrophils, lymphocytes, monocyte/macrophages) in BALF of cSiO_2_/P0 as compared to VEH-instilled mice fed CON diet (**Supplementary Figure 7**). PL and PM consumption did not attenuate cSiO_2_-induced increases of inflammatory cell numbers in the BALF. Interestingly, lymphocytic cell numbers significantly increased in BALF of the cSiO_2_/PM group as compared to the cSiO_2_/P0 animals.

IHC and morphometry were used to further characterize ELS lymphoid cell populations affected by cSiO_2_ and prednisone treatments. PM but not PL consumption significantly reduced cSiO_2_-induced CD45R^+^ B-cell accumulation in pulmonary perivascular and peribronchiolar regions (**Figure 3B**) but neither treatment affected cSiO_2_-triggered CD3^+^ T-cell infiltration (**Figure 3C**). PM consumption modestly reduced cSiO_2_-induced increases in pulmonary IgG^+^ plasma cell density (**Supplementary Figure 8A**), but not overall IgG deposition (**Supplementary Figure 8B**), whereas PL diet had no effect on either of these responses (**Supplementary Figures 8A, B**).

### Prednisone intake selectively inhibited cSiO_2_-induced nuclear AAbs in the BALF

**Figure 4:**
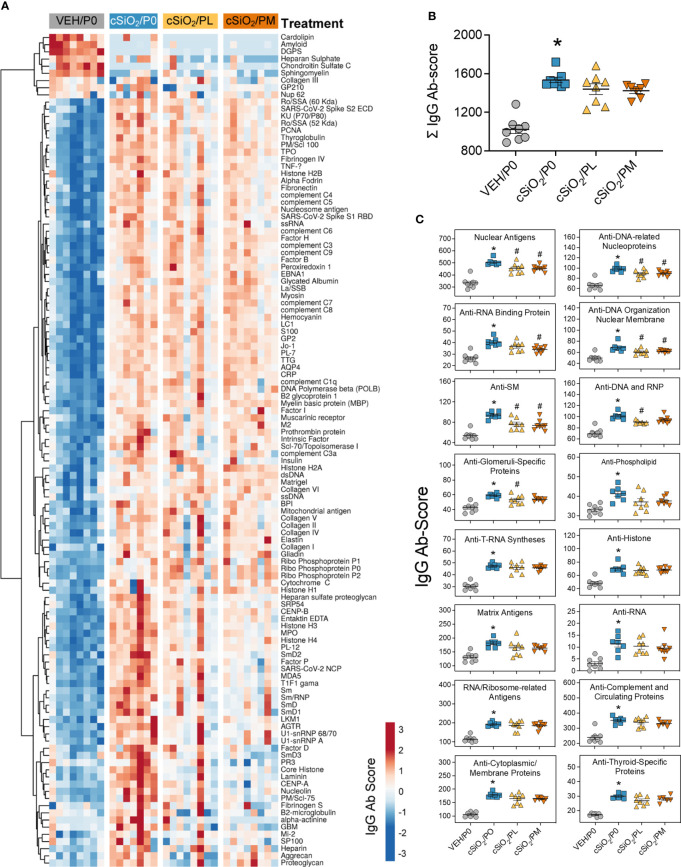
Prednisone treatment is effective in reducing a subset of cSiO_2_-induced AAbs in the BALF. AAbs production was measured in BALF using Cohort A samples collected at time of necropsy (14 wk PI). **(A)** Heat map illustrates unsupervised clustering (Euclidian distance method) of 122 AAbs shown as Ab-score values for IgG expression in BALF. Scale bar values reflect the range of variance-stabilized Ab scores, which were centered across rows. **(B)** Prednisone does not significantly reduce cSiO_2_-triggered increased total IgG levels in the BALF. **(C)** prednisone treatment was effective in significantly reducing certain classes of autoantibodies in the BALF compared to cSiO_2_/P0 positive control. *Indicates p<0.05 for VEH/P0 vs cSiO_2_/P0; ^#^indicates p<0.05 for cSiO_2_/P0 vs cSiO_2_/PL or cSiO_2_/PM group.

Consistent with our prior reports, AAg microarray analysis indicated cSiO_2_ instillation triggered significant increases in IgG AAbs in both the BALF (**Figure 4A**) and plasma (**Supplementary Figure 9A**) of cSiO_2_/P0 mice compared to VEH/P0 mice, which was further reflected in AAb summation scores (**Figure 4B** **;** **Supplementary Figure 9B**). Prednisone consumption did not significantly reduce AAb summation scores in the BALF and plasma; however, PL and PM diets were effective at reducing AAbs specific for nuclear AAgs detected in the BALF (**Figures 4A, C**) but not the plasma (**Supplementary Figure 9C**).

### Moderate prednisone consumption reduced cSiO_2_-induced splenic lymphoid hyperplasia and splenomegaly

**Figure 5:**
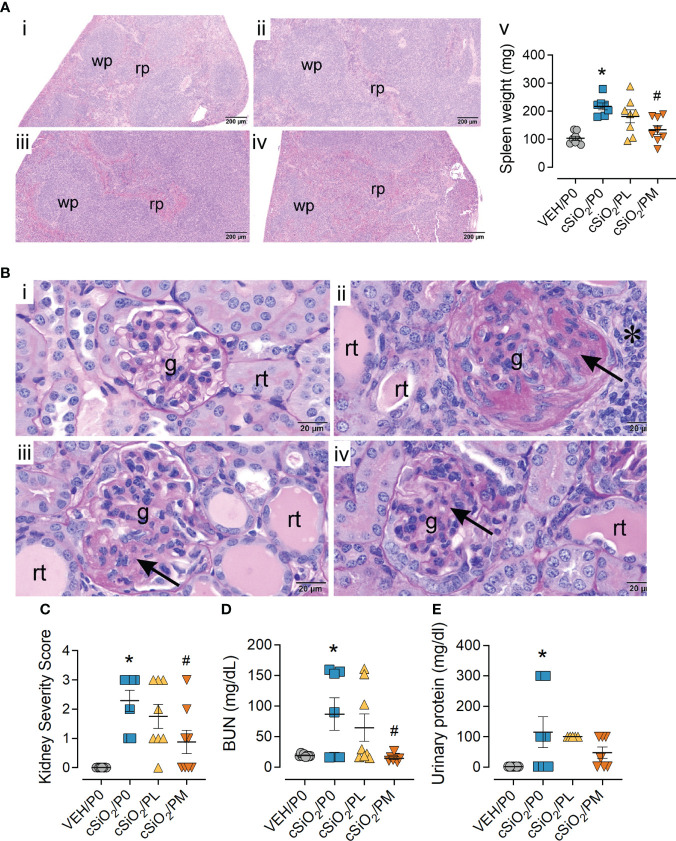
Moderate dose prednisone (PM) significantly reduced cSiO_2_-induced splenomegaly and glomerulonephritis. **(A)** Light photomicrographs of hematoxylin and eosin-stained splenic tissue and **(B)** periodic acid Schiff (PAS) and hematoxylin-stained renal tissue from **(i)** VEH/P0, **(ii)** cSiO_2_/P0, **(iii)** cSiO_2_/PL, and (iv) cSiO_2_/PM mice. Splenic white pulp (wp) is increased (lymphoid cell hyperplasia) in A.ii and A.iii compared to A.i. WP is similar in VEH/P0 (A.i) and cSiO_2_/PM (A.iv) mice. Prednisone inhibited cSiO_2_-induced increases in spleen weight (A.v) Glomeruli (g) in B.ii and B.iii are enlarged and hypercellular with PAS-stained membranous tissue (arrows). Dilated renal tubules (rt) with luminal proteinaceous material are present in B.ii and B.iii, but not in B.i. PM treatment results in modest glomerular and renal tubular histopathology in B.iv. **(C)** Individual kidney sections were semi-quantitatively scored based on the modified International Society of Nephrology/Renal Pathology Lupus Nephritis Classification system described methods for lupus nephritis score. **(D)** PM treatment significantly reduced cSiO_2_-induced plasma BUN at time of necropsy **(E)** but was ineffective in significantly reducing urinary protein assessed at 12 wks PI. Statistical analyses were performed as described in Methods. *Indicates p<0.05 for VEH/P0 vs cSiO_2_/P0; ^#^indicates p<0.05 for cSiO_2_/P0 vs cSiO_2_/PL or cSiO_2_/PM group.

cSiO_2_/P0 mice exhibited extensive lymphoid hyperplasia in the splenic white pulp that was not observed in the VEH/P0 group (**Figures 5A.i, ii**). Hyperplasia was less prominent in cSiO_2_/PL mice (**Figure 5A.iii**) and conspicuously less so in cSiO_2_/PM mice (**Figure 5A.iv**). Correspondingly, cSiO_2_/P0 exhibited higher spleen weights than VEH/P0 mice and, furthermore, PM treatment suppressed cSiO_2_-induced splenic weight increases (**Figure 5A.v**).

### Moderate prednisone intake inhibited cSiO_2_-induced glomerulonephritis

Consistent with previous studies (13, 19), histopathological analysis revealed cSiO_2_ induced glomerulonephritis and protein accumulation in kidneys of cSiO_2_/P0 mice compared to VEH/P0 mice (**Figures 5B.i, ii**). These responses were significantly reduced by PM intake (**Figure 5B.iv**). Likewise, the PM diet significantly reduced cSiO_2_-induced increases in kidney severity scores (**Figure 5C**). cSiO_2_ treatment resulted in a significant increase in plasma BUN levels (**Figure 5D**) and proteinuria (**Figure 5E**). PM treatment was effective in significantly reducing plasma BUN values but not protein levels in the urine.

### Moderate prednisone intake influenced cSiO_2_-induced immune pathway transcriptional changes in lung and kidney tissues

**Figure 6:**
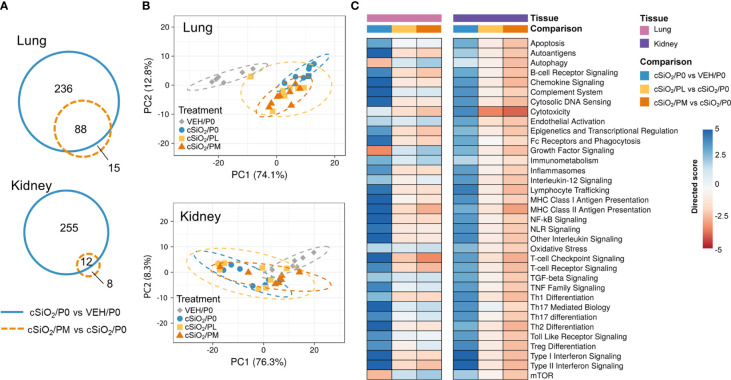
Effect of prednisone treatment on cSiO_2_-induced transcriptional changes in lung and kidney tissues of mice 14 weeks post instillation. **(A)** Venn diagrams depicting overlap of genes differentially regulated in mice with cSiO_2_/P0 vs VEH/P0 treatment or cSiO_2_/PM vs cSiO_2_/P0 treatment (FDR q<0.05, 1.5-fold change). The overlap regions indicate genes affected by silica exposure that were also differentially regulated with moderate-dose prednisone. No significant differentially expressed genes were identified when comparing cSiO_2_/PL to cSiO_2_/P0 for either lung or kidney. **(B)** Principal components analyses of differentially expressed genes in lung and kidney tissues of mice with low- (cSiO_2_/PL) or moderate-dose prednisone (cSiO_2_/PM) as compared to VEH/CON and cSiO_2_-exposed (cSiO_2_/P0) tissue-matched control diets. PC1 and PC2 are shown with 95% confidence intervals (dashed ellipses). **(C)** Directed significance scores for autoimmune pathways were determined using nSolver (see Materials and Methods) by comparing cSiO_2_/P0 to tissue-matched VEH/P0 control group or by comparing cSiO_2_/PL or cSiO_2_/PM treatments to tissue matched cSiO_2_/P0 treatment group. cSiO_2_, crystalline silica; P0, zero prednisone; PL, low-dose prednisone; PM, moderate-dose prednisone; VEH, vehicle control.

NanoString analysis revealed that, in the lung, cSiO_2_ exposure resulted in 324 differentially expressed genes and 103 differentially expressed genes with PM treatment. In the kidney, cSiO_2_ induced 267 differentially expressed genes in P0-fed mice and 20 in PM-fed mice (**Figure 6A**). Principal component analyses (PCA) showed distinct gene profiles between VEH/P0 and cSiO_2_/P0 mice in both lung and kidney tissues (**Figure 6B**). Gene profiles were less distinguishable between cSiO_2_/P0 and cSiO_2_/PM groups in both tissues.

Immunological pathways significantly affected by cSiO_2_ and prednisone treatments were identified based on global and directed significance scores (**Figure 6C**). cSiO_2_ exposure led to broad activation of immune pathways in both the lung and kidney. Alternatively, repression of certain nutrient sensing (autophagy and mTOR) and growth factor signaling pathways occurred in the lung, specifically, in response to cSiO_2_. Prednisone treatment at both low and moderate doses modestly reduced cSiO_2_-induced effects on most immune pathways in both tissues. PM treatment demonstrated greater inhibition of cSiO_2_-stimulated pathways such as those pertaining to cytotoxicity, MHC Class II antigen presentation, T-cell checkpoint signaling, and T-cell receptor signaling. These findings corresponded with calculated Z scores derived from expression values for specific genes assigned to representative immune pathways in the lung (**Supplementary Figure 10A**) and kidney (**Supplementary Figure 10B**). In the lung, network mapping revealed genes associated with innate and adaptive immunity (interleukins, cytokines, IFN), lymphocyte and macrophage function (antigen presentation and MHC), complement, and nuclear proteins were significantly affected by PM treatment (**Supplementary Figure 10C**). Genes associated with inflammation (interleukins, chemokines, cytokines), cell adhesion, apoptosis, lymphocyte and macrophage function (antigen presentation and MHC), and endothelial cell function were most affected by PM treatment in the kidney (**Supplementary Figure 10D**).

### Prednisone modulated cSiO_2_-induced autoimmune-related gene expression in lung and kidney tissues

**Figure 7:**
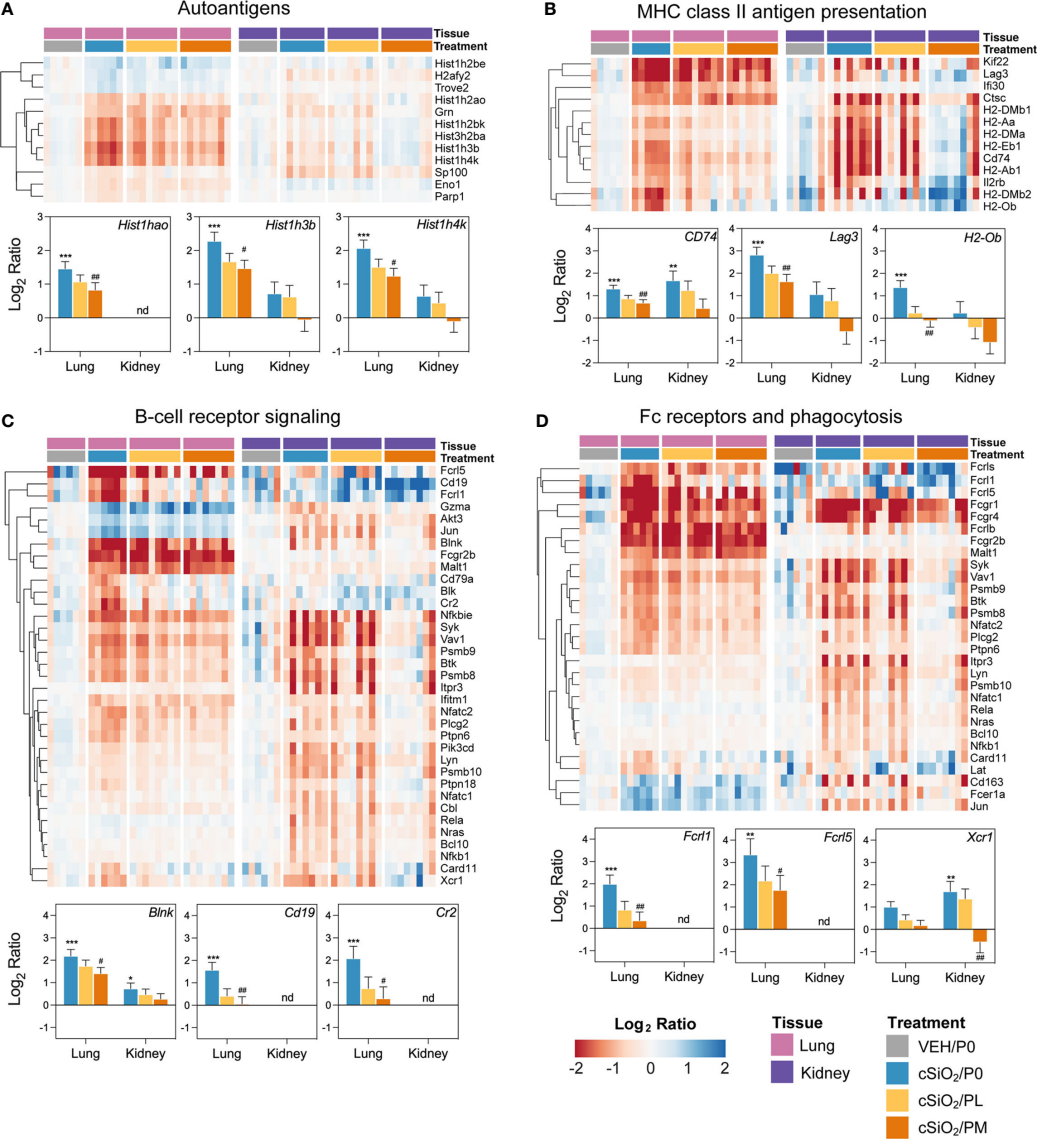
Comparison of prednisone-responsive genes associated with **(A)** autoantigen, **(B)** MHC class II antigen presentation, **(C)** B-cell receptor signaling, and **(D)** Fc receptors and phagocytosis pathways in lung or kidney tissues 14 weeks post instillation with cSiO_2_. Gene expression data were obtained using the NanoString Autoimmune Profiling gene panel and are shown as log2 ratios for cSiO_2_/P0, cSiO_2_/PL, and cSiO_2_/PM treatment groups with respect to the tissue-matched VEH/P0 control group (log2 ratio = 0). For each pathway, heatmaps with unsupervised hierarchical clustering (Euclidian distance method) by gene show log2 expression values for all genes identified as differentially expressed in response to either cSiO_2_ exposure or moderate-dose prednisone (FDR q<0.05, 1.5-fold change) in either of the selected tissues. The mean log2 ratio values + SEM for selected genes of interest are also shown for each pathway. For for cSiO_2_/P0 as compared to VEH/P0, *, FDR-corrected q<0.05; **, q<0.01; and ***, q<0.001. For cSiO_2_/PL or cSiO_2_/PM vs cSiO_2_/P0, ^#^, FDR-corrected q<0.05; and ^##^, q<0.01. nd stands for not detected. See **Supplementary File 1** for test specifications and FDR-corrected q-values for all genes in the panel for all comparisons. cSiO_2_, crystalline silica; P0, zero prednisone; PL, low-dose prednisone; PM, moderate-dose prednisone; VEH, vehicle control.

**Figure 8:**
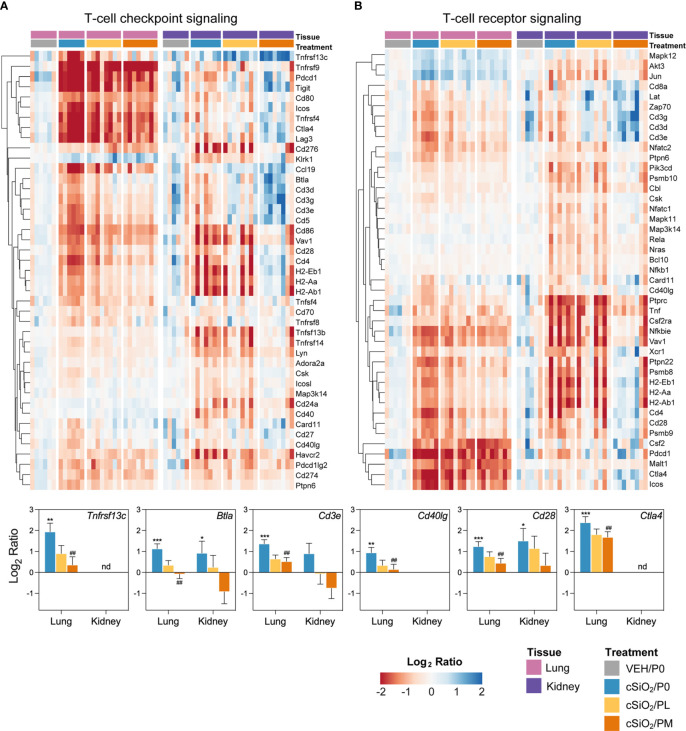
Comparison of prednisone-responsive genes associated with **(A)** T-cell checkpoint signaling and **(B)** T-cell receptor signaling pathways in lung or kidney tissues 14 weeks post instillation with cSiO_2_. Gene expression data were obtained using the NanoString Autoimmune Profiling gene panel and are shown as log2 ratios for cSiO_2_/P0, cSiO_2_/PL, and cSiO_2_/PM treatment groups with respect to the tissue-matched VEH/P0 control group (log2 ratio = 0). For each pathway, heatmaps with unsupervised hierarchical clustering (Euclidian distance method) by gene show log2 expression values for all genes identified as differentially expressed in response to either cSiO_2_ exposure or moderate-dose prednisone (FDR q<0.05, 1.5-fold change) in either of the selected tissues. The mean log2 ratio values + SEM for selected genes of interest are also shown. For cSiO_2_/P0 as compared to VEH/P0, *, FDR-corrected q<0.05; **, q<0.01; and ***, q<0.001. For cSiO_2_/PL or cSiO_2_/PM vs cSiO_2_/P0, ##, FDR-corrected q<0.01. nd stands for not detected. See **Supplementary File 1** for test specifications and FDR-corrected q-values for all genes in the panel for all comparisons. cSiO_2_, crystalline silica; P0, zero prednisone; PL, low-dose prednisone; PM, moderate-dose prednisone; VEH, vehicle control.

Heatmaps and bar graphs of representative genes from cSiO_2_-impacted autoimmune pathways illustrate those most highly influenced by prednisone treatment (**Figures 7**, **8** **;** **Supplementary Figure 11**). In accordance with intranasal cSiO_2_ exposure, the expression of genes coding for nuclear material (*e.g., Hist1hao, Hist1h3b, Sp100*) and associated with AAb production were upregulated in the lung of cSiO_2_/P0 mice compared to VEH/P0 mice (**Figure 7A**). Consistent with previous studies (17, 41), cSiO_2_ upregulated genes contained in pathways such as MHC II antigen presentation (**Figure 7B**), B-cell signaling (**Figure 7C**), Fc receptors and phagocytosis (**Figure 7D**), lymphocyte tracking (**Supplementary Figure 11A**), oxidative stress (**Supplementary Figure 11B**), cytotoxicity (**Supplementary Figure 11C**), and T-cell checkpoint and receptor signaling (**Figures 8A, B**) in both the lung and kidney. PM treatment markedly reduced some cSiO_2_-induced autoimmune-related gene expression in the lung and kidney tissues while others remained upregulated (**Figures 7**, **8** **;** **Supplementary Figure 11**).

**Figure 9:**
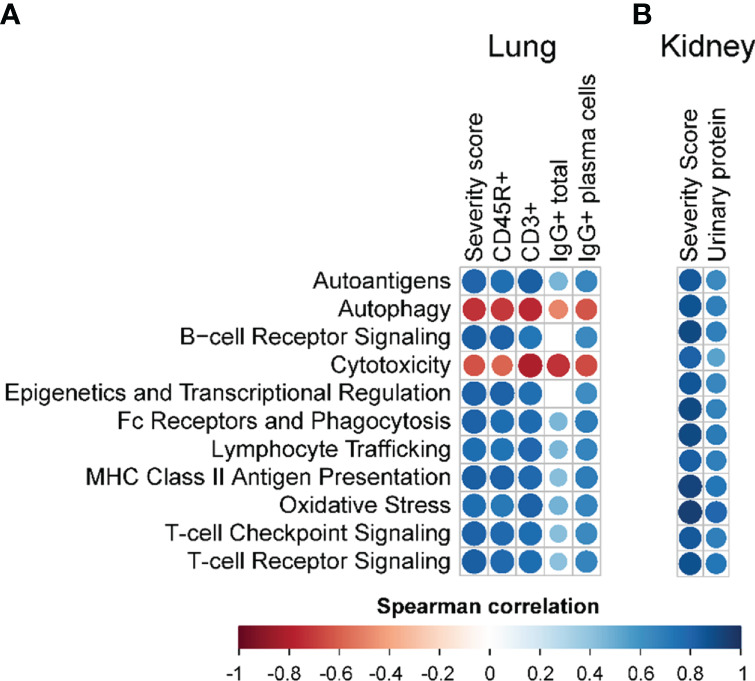
Correlation analyses of selected autoimmune pathways. For all treatment groups, spearman ρ values were calculated by correlating pathway Z scores with **(A)** lung severity score or the percent positive staining for CD45R^+^, CD3^+^, or IgG^+^ in lung tissue or IgG^+^ in lung tissue plasma cells; or **(B)** kidney severity score, kidney blood urea nitrogen (BUN), or kidney urinary protein. Significant correlation values (p<0.05) are represented as circles colored by the correlation value (blue, positive; red, negative); non-significant correlations are indicated by blank cells.

Immune pathways enriched due to cSiO_2_ exposure, and conversely suppressed by PM treatment, correlated with pathological endpoints evaluated in the lung and kidney (**Figure 9** **;** **Supplementary Figure 12**). Immune cell type profiling, which was determined based on gene expression data to identify various cell types, revealed significant increases in B- and T-cell infiltration in the lung following cSiO_2_ exposure (**Supplementary Figure 13A**). Interestingly, B-cell but not T-cell morphometric analysis (**Figures 3B.v, C.v**) was consistent with PM-induced decreases in immune cell scoring. cSiO_2_ exposure caused B- and T-cells to comprise a large fraction of total infiltrating leukocytes (TILs), which was reduced with PM administration (**Supplementary Figure 13C**). In contrast, PL and PM treatment led to an increase in the fraction of macrophages that comprise TILs. In the kidney, cSiO_2_ led to significant increases in cytotoxic cells and macrophages (**Supplementary Figure 13B**). Scoring for cytotoxic cells was significantly decreased with PM administration. Prednisone had negligible influence on cSiO_2_-induced changes in cell fractions of TILs (**Supplementary Figure 13D**). When considering overall TILs, PM treatment effectively suppressed cSiO_2_-triggered increases in both the lung and kidney tissues (**Supplementary Figures 13C, D**).

### cSiO_2_ and prednisone had modest effects on blood mRNA signatures

NanoString profiling of mRNA isolated from whole blood samples revealed limited effects on immune pathway enrichment and cell type scoring when comparing VEH/P0 vs cSiO_2_/P0 and cSiO_2_/P0 vs cSiO_2_/PL or PM groups (**Supplementary Figures 14–18**). Modest cSiO_2_-induced transcriptomic changes were observed in blood taken 11 wk PI, whereas prednisone-modulated genes were observed primarily at 7 wk PI (**Supplementary Figure 15A**). cSiO_2_ exposure had a significant effect on genes involved in lymphocyte trafficking and stimulation (e.g., *CD3, IL-17r, Ccr7*) and interferon response (e.g., *Irf7, Ifitm3*) (**Supplementary Figure 16**). Similar to what was observed in lung and kidney tissues, genes related to antigen presentation (e.g., *H2-Ob, H2-Dmb2*) and B- and T-cell signaling (e.g., *Cd19, Cd74, Btla*) were significantly modulated by moderate prednisone compared to cSiO_2_/P0 mice (**Supplementary Figures 16B, 17A, B**). However, it is important to note cSiO_2_ did not significantly upregulate these genes compared to VEH/P0 mice at this same timepoint. At 11 wk PI, cSiO_2_ significantly upregulated genes that factored into immune cell scores for macrophages and neutrophils while decreasing those for cytotoxic cells and T-cells (**Supplementary Figure 18A**). The scoring of these individual immune cell types corresponded with their respective fraction of total infiltrating leukocytes (TILS) (**Supplementary Figure 18B**). Only cytotoxic and T-cell fractions of TILs were modulated by PM treatment at this timepoint. Taken together, these data suggest that, unlike in the lung and kidney, the effects of cSiO_2_ on inflammatory and autoimmune mRNA expression in whole blood were minimal, and marginally impacted by prednisone.

## Discussion

Major goals in lupus treatment are to achieve drug-free remission in patients, where there is little to no disease activity for 5 or more years, and to slow the progression of organ damage (46). Often, patients with lupus are given bolus intravenous doses of prednisolone, the active metabolite of prednisone, ranging from 250-1000 mg/d for 3 days to provide quick relief from flaring through non-genomic pathways before starting a tapering regimen of prednisone to maintain inhibition of the inflammation (2, 20, 47). Unfortunately, there are inconsistencies when it comes to tapering and questions surrounding how much prednisone is needed to help patients achieve remission. Thus, tapering regimens can range from lower doses of ≤7.5 mg/d, to medium doses of >7.5-30 mg/d, and finally higher doses of 30-100 mg/d (21). The investigation described herein is the first to evaluate the dose-dependent response of prolonged prednisone dietary administration on the induction of GC toxicity and the onset/progression of environmental-triggered autoimmunity in a preclinical lupus mouse model. Several novel observations were made. First, we demonstrated that dietary incorporation is a simple and efficient way to deliver clinically relevant prednisone doses to rodents. Second, we found that PH caused significant weight loss, muscle wasting, glucosuria, and mortality, thus necessitating early removal of this experimental group from the study. PM but not PL intake caused muscle wasting and weight loss; however, neither treatment influenced urinary glucose or bone density. Third, PM but not PL was effective in reducing cSiO_2_-triggered peribronchiolar and perivascular ELS neogenesis, B- and plasma-cell accumulation, and AAb production against nuclear related autoantigens in the lung. However, neither moderate nor low dose prednisone affected cSiO_2_-induced macrophage, neutrophil, or lymphocyte increases in the BALF, pulmonary and perivascular T-cell accumulation, or plasma AAb responses. Fourth, PM but not PL attenuated cSiO_2_-induced glomerulonephritis and plasma BUN. Fifth, consistent with the pathological findings, PM significantly reduced cSiO_2_-triggered gene expression in several inflammation- and autoimmunity-associated pathways in both lung and kidney tissues. Finally, despite the significant inhibition of autoimmunity and glomerulonephritis, PM did not prolong survival of cSiO_2_-treated mice. Therefore, while GC-associated toxicity endpoints from this study are important to consider, PM was effective in attenuating cSiO_2_-triggered ELS formation in the lung which importantly serves as the nexus for downstream autoimmunity and resultant glomerulonephritis.

Despite the wide usage of prednisone to manage lupus, there have been limited investigations regarding its therapeutic and toxic properties in preclinical models. These studies have varied in terms of mouse strain, duration of exposure, and route of administration. Relevant to the present study, the lupus-prone MRL/lpr mouse strain has been used to evaluate suppression of spontaneous autoimmunity following intragastric administration of a prednisone solution daily for 13 weeks (48) or oral administration of a prednisolone solution daily for 4 weeks (49). Alternatively, others have used non-lupus-prone strains such as Crl : CD-1(ICR) (35), C57BL/6 (50), or Dmd^mdx^ (46) mice to evaluate prednisone or prednisolone toxicity in the context of carcinogenicity, insulin resistance, and muscle wasting, respectively. While these studies have provided valuable insights into the anti-inflammatory and toxic effects of oral prednisone and prednisolone exposure, none have assessed both outcomes simultaneously as done here.

A central question arising from our use of prednisone-amended diets relates to their relevance to human intake of the drug in a clinical setting. Oral prednisone has a half-life of 3 to 4 h in humans (45) and is typically prescribed in an immediate release form as a single daily dose or 3 to 4 divided doses/day, or, alternatively, in a delayed release form taken once per day (48). When mice are housed under a standard 12 h light/12 h dark cycle, as employed in this study, nearly all their food consumption occurs during the dark cycle, with short episodic feeding occurring during the light cycle (49). Thus, most prednisone ingestion likely occurs during the dark feeding cycle, mimicking intake of the immediate release form of the drug in divided doses with meals throughout the day. Importantly, we found the mice efficiently converted prednisone consumed *via* the diet to its biologically active metabolite, prednisolone. Mean prednisolone concentrations in blood of mice fed PL, PM, and PH diets were 27, 105, 151 ng/ml, respectively. These results compare favorably to those of Rose et al. (45), who gave 5, 20, or 40 mg oral prednisone to healthy human males and observed plasma concentrations of prednisolone between 10-40 ng/ml, 24-80 ng/ml, and 75-200 ng/ml, respectively between 6 and 12 h post-administration. In another study (47), adolescent and young adult patients with lupus were given between 5 and 40 mg of prednisone (mean ± SEM =19.5 ± 11.5) their average prednisolone concentrations were 200, 150, 90, and 50 ng/ml at 2, 4, 6, and 9 h, respectively. Recently, Mangin et al. (51) assessed prednisone pharmacokinetics in 107 patients with inflammatory/autoimmune diseases following oral dosing with 5 to 20 mg prednisone and reported 50th percentile levels of approximately 300, 150, and 50 ng/ml at 2, 5, and 9 h after dosing, respectively. Collectively, these reports support the contention that prednisolone concentrations stably attained in blood through feeding prednisone diets in our model are highly consistent with those that might be expected in humans given therapeutic doses of this prodrug.

Consistent with previous studies from our lab, repeated intranasal exposure to cSiO_2_ beginning at 8-wks of age was effective in initiating unresolved inflammation in the lung leading to a loss of self-tolerance and the development of glomerulonephritis (13, 14, 16, 19). Repeated cSiO_2_ exposure also led to the production of AAbs corresponding to certain nuclear antigens such as SM, RNP, histone, and DNA-related nucleoproteins which have been commonly implicated in lupus (1, 52, 53). The extent to which prednisone effectively reduced cSiO_2_-induced inflammation, as demonstrated by the pathological findings, was consistent with mRNA signatures in the lung and kidney. NanoString analysis showed that cSiO_2_-responsive genes differed based on whether the tissue had direct contact with the cSiO_2_ particles (lung) or was responding to the systemic autoimmunity initiated by AAbs and immune complexes (kidney). Pro-inflammatory gene expression initiated by cSiO_2_ exposure was effectively brought down with the moderate dose of prednisone. Prednisone demonstrated modest efficacy in suppressing cSiO_2_-induced AAb production detected in the BALF, as it only significantly reduced pathogenic AAbs specific to nuclear and glomeruli-specific antigens. These data coincide with our NanoString analysis where many of the prednisone-responsive genes under the autoantigen and MHC class II antigen presentation pathways in the lung were specific to nuclear material (e.g., *Hist1hoa, Hist1h3b, H2-Ob*). Immune cell type enrichment using nSolver analysis indicated cSiO_2_-dependent increases in macrophages and B-cells in the lung, similar to what was observed *via* differential cell counts in the BALF and morphometric analysis, respectively. Interestingly, lymphocyte numbers detected in the BALF were significantly increased with moderate prednisone treatment compared to cSiO_2_ control mice. This suggests that prednisone may exacerbate the upregulation of certain lymphoid cell populations elicited by cSiO_2_. Additionally, prednisone did not reduce overall pulmonary CD3^+^ T-cell counts as determined with morphometry but did significantly reduce T-cell counts in immune cell profiling using NanoString. It is possible that prednisone promoted T-cell differentiation and replaced inflammatory T-cell populations with those involved in maintaining peripheral tolerance. It has been reported by others that GCs inhibit the differentiation of proinflammatory T-helper cells and enhance the differentiation of anti-inflammatory T-reg cells (54), which could explain the high number of T-cells still present with prednisone treatment in immunohistochemically stained tissues. However, more research is necessary to explain prednisone’s preferential inhibition of pulmonary B-cell rather than T-cell recruitment in our model.

It is well-documented in the literature that prolonged use of GCs such as prednisone can lead to many deleterious side-effects such as cardiovascular disease, osteoporosis, myopathy, diabetes, and increased risk of secondary infection (20–24). It has been suggested that prednisone doses >30-40 mg/d cease to serve any further anti-inflammatory benefits due to saturation of the GC genomic pathway, and this dose range is also the threshold for undesirable side-effects resulting from GC transactivation pathways (21). This can be extremely problematic for lupus patients considering it is extremely rare for them to achieve drug-free remission (46). Only 3-7% of lupus patients can achieve drug-free remission (51). Additionally, physicians are reported to be more likely to keep patients on GCs indefinitely if there is evidence of past organ involvement despite decreases in disease activity (20). Due to the unlikelihood of lupus patients attaining drug-free remission, they will likely rely on GCs for the remainder of their lives, putting them at greater risk for GC-induced toxicity (25). Therefore, lower doses of ≤7.5 mg/d have been increasingly recommended as the standard for tapering (20, 21, 46). Our findings that the PL diet (≈5 mg/day HED) was not toxic but the PM (≈14 mg/d HED) and PH (≈46 mg/d HED) diets were toxic, is consistent with the 7.5 mg/d clinical threshold.

In a study by Yan et al. (48) using MRL/lpr lupus prone mice, prednisone administered intragastrically at doses of 2.5 and 5 mg/kg/d for 13 wks significantly reduced spontaneous production of anti-nuclear AAbs, but not anti-dsDNA AAbs in the serum. Even though we did not observe the same prednisone-induced reduction of AAbs in the systemic circulation, it is interesting to note that those investigators also saw inhibition of anti-nuclear AAbs specifically, similar to the AAb classes affected in our BALF samples. It is also possible that we did not see similar anti-nuclear AAb suppression in the systemic circulation due to our use of lower doses of prednisone.

A central finding of this study is that prednisone treatment had collateral toxic effects and did not have any impact on plasma AAbs or life expectancy of cSiO_2_-treated lupus-prone mice. These findings highlight the critical need for identifying alternative steroid-sparing therapeutics to improve the quality of life in lupus patients. Towards this goal, we have found in prior studies that diets enriched with clinically relevant doses of the omega-3 fatty acid, docosahexaenoic acid (DHA) were highly efficacious in preventing and/or treating cSiO_2_-induced autoimmunity in the female NZBWF1 mouse (14, 16–19). Unlike prednisone, DHA was effective in reducing both cSiO_2_-induced B- and T-cell infiltration in the lung. Additionally, DHA was effective in significantly reducing cSiO_2_-triggered pathogenic AAb in the plasma along with a broader repertoire of AAbs in the BALF compared to prednisone. While prednisone effectively reduced some AAbs in the BALF that are associated with lupus (Smith antigens), DHA also suppressed most of those linked to other autoimmune diseases such as arthritis (fibrinogen), vasculitis (MPO, prednisone3), and myositis (MDA5, Mi-2) (18, 55).

Overall, the toxic effects of prednisone outweighed its ameliorative effects on lupus disease which is why there was no improvement in survival. While it was not within the scope of the present study to do a thorough investigation as to why prednisone-fed mice without improved survivability, incomplete suppression of systemic autoimmunity is a likely factor. While prednisone was effective in preventing inflammation in the kidney, it was not effective in significantly reducing proteinuria or pathogenic autoantibodies in the systemic circulation. So, it is probable that incomplete protection of prednisone outside of the lung coupled with weight/muscle loss contributed to decreased survival.

One limitation of this investigation is that since the large dose of cSiO_2_ used here is poorly cleared from lung after instillation, it causes unresolved inflammation that might overwhelm the ameliorative effects of oral prednisone. It will therefore be useful in future studies to ascertain the dose response effects of dietary prednisone in the context of slower, spontaneous development of autoimmunity and glomerulonephritis in female NZBWF1 mice and other lupus-prone mouse strains. Another limitation of this study is that it focused on the preventive effects of prednisone rather than more clinically relevant therapeutic intervention after autoimmunity onset. For example, is possible that short term treatment with high dose prednisone for 1 or 2 wk after cSiO_2_ dosing followed by a tapering regimen might be effective at reducing cSiO_2_-triggered autoimmunity with minimal GC-associated toxicity.

Taken together, this preclinical study demonstrates that prolonged dietary administration of prednisone at clinically relevant doses falls short of offering complete protection from cSiO_2_-induced autoimmunity and can cause significant secondary toxicity at moderate to high doses. While it is unclear why prednisone-fed mice without improved survivability, we can conclude that secondary GC toxicity likely outweighed its protective effects which were mainly localized to the lung. Nevertheless, prednisone’s ability to quell inflammation stemming from ELS formation in the lung provides novel insights important to the utilization of prednisone in human lupus triggered by inhaled toxicants. Our findings from this and prior studies highlight the value of this preclinical model for investigating alternative safe, cost-effective therapies for lupus, such as omega-3 fatty acid supplementation, which can potentially lower requisite prednisone doses needed to reduce inflammation and suppress autoimmunity thereby improving the quality of life of lupus patients.

## Data availability statement

The data presented in the study are deposited in the Dryad repository and can be found using the link: https://doi.org/10.5061/dryad.2280gb5vx.

## Ethics statement

The animal study was reviewed and approved by Michigan State University Institutional Animal Care and Use Committee (AUF #PROTO201800113).

## Author contributions

LH: study design, coordination, feeding study, necropsy, data curation, data analysis/interpretation, morphometrical analysis, figure preparation, manuscript preparation and submission. AB: data acquisition/analysis, figure preparation, manuscript writing. ER: feeding study, mouse handling, data acquisition. LR: data acquisition/analysis, figure preparation, manuscript writing. JW: necropsy, lab analysis. RL: instillations, necropsy, lab analysis. Q-ZL: microarray. JB and JZ: LC-MS/MS, data analysis, manuscript writing. AR: mouse handling and feeding, data acquisition. AT: morphometric analysis. NC and LM: Micro-CT and manuscript preparation. JH: study design, oversight, lung/kidney histopathology, morphometry, data analysis, manuscript preparation. JP: study design, oversight, funding acquisition, lung/kidney histopathology, morphometry, data analysis, manuscript preparation and submission. All authors contributed to the article and approved the submitted version.

## Funding

This research was funded by NIH T32ES007255 (LH), NIH ES027353 (JP), Lupus Foundation of America (JP), Albert C. and Lois E. Dehn Endowment at Michigan State University (JH), and Dr. Robert and Carol Deibel Family Endowment (JP).

## Acknowledgments

First, the authors thank Amy Porter of the Michigan State University Laboratory for Investigative Histopathology for their assistance with the histotechnology. Second, they thank Leslie Pittsley and Michigan State University’s Campus Animal Resources for their assistance with blood collections. Lastly, they thank Dr. Kevin Childs and the Research Technology Support Genomics Core Facility for processing samples using NanoString nCounter Analysis System.

## Conflict of interest

The authors declare that the research was conducted in the absence of any commercial or financial relationships that could be construed as a potential conflict of interest.

## Publisher’s note

All claims expressed in this article are solely those of the authors and do not necessarily represent those of their affiliated organizations, or those of the publisher, the editors and the reviewers. Any product that may be evaluated in this article, or claim that may be made by its manufacturer, is not guaranteed or endorsed by the publisher.
