# Supplementary material for: Comparative effects of human-equivalent low, moderate, and high dose oral prednisone intake on autoimmunity and glucocorticoid-related toxicity in a murine model of environmental-triggered lupus
Source: Front Immunol. 2022 Oct 19;13:972108. doi: 10.3389/fimmu.2022.972108 (PMC9627297; doi:10.3389/fimmu.2022.972108)
Supplement: Supplementary file 3 [file DataSheet_3.pdf]

frontiers in Immunology

Supplementary Material

Comparative Effects of Human-Equivalent Low, Moderate, and High Dose Oral Prednisone Intake on Autoimmunity and Glucocorticoid-Related Toxicity in a Murine Model of Environmental-Triggered Lupus

Lauren K. Heine[1,2], Abby D. Benninghoff[3*], Elizabeth A. Ross[4], Lichchavi D. Rajasinghe[4], James G. Wagner[2,5], Ryan P. Lewandowski[5], Alexa L. Richardson[4], Quan-Zhen Li[6], John P. Buchweitz[2,5,7], Justin Zyskowski[7], Ashleigh N. Tindle[5], Anna E. Skedel[5], Nicholas J. Chargo[8], Laura R. McCabe[8], Jack R. Harkema[1,2,5], and James J. Pestka[2,4,9*]

[1]Department of Pharmacology and Toxicology, Michigan State University, East Lansing, MI 48824, U.S.
[2]Institute for Integrative Toxicology, Michigan State University, East Lansing, MI 48824, U.S.
[3]Department of Animal, Dairy and Veterinary Sciences, School of Veterinary Medicine, Utah State University, Logan, UT, U.S.
[4]Department of Food Science and Human Nutrition, East Lansing, MI 48824, U.S.
[5]Department of Pathobiology and Diagnostic Investigation, Michigan State University, East Lansing, MI 48824, U.S.
[6]Department of Immunology and Internal Medicine, IIMT Microarray Core Facility, University of Texas Southwestern Medical Center, Dallas, TX, U.S.
[7]Toxicology Section, Michigan State University Veterinary Diagnostic Laboratory, Lansing, MI 48910, U.S.
[8]Department of Physiology, Michigan State University, East Lansing, MI 48824, U.S.
[9]Department of Microbiology and Molecular Genetics, Michigan State University, East Lansing, MI 48824, U.S.

*** Correspondence:**
Abby D. Benninghoff, Department of Animal, Dairy and Veterinary Sciences, School of Veterinary Medicine, Utah State University, Logan, UT, U.S. Email: abby.benninghoff@usu.edu;
James J. Pestka, Department of Food Science and Human Nutrition, Michigan State University, East Lansing, MI 48824, U.S. E-mail: pestka@msu.edu

Materials and Methods for Prednisone and Prednisolone Measurement

Chemicals and reagents

Prednisone was obtained from Sigma Aldrich (St. Louis, MO). Prednisolone and deuterated prednisone (*d*7) were obtained from Toronto Research Chemicals (Toronto, Canada). Reagent grade acetonitrile was obtained from Fisher Scientific (Waltham, MA). Mass spectrometry grade formic acid (>98%) was obtained from Merck. Deionized water was provided by a Milli-Q Ultrapure Water System from Millipore Corporation (Bedford, MA).

Standards and Calibrants

Drug-free bovine serum was used for developing matrix matched standards for serum analysis. A portion of drug-free feed was used for matrix specific standard addition analysis. Prednisone and prednisolone were prepared as neat standards in acetonitrile to concentrations of 1 mg/mL. Feed standards were prepared by spiking prednisone across the study-defined working range (0, 5, 20, 100 μg/g feed) with internal standards added (25 μg/g Prednisone-*d*7). Serum standards were prepared by spiking prednisolone across the study-defined working range (0, 1, 5, 10, 20, 50, 100, 200 ng/mL serum) with internal standards added (10 ng/mL Prednisone-*d*7). The relative peak areas for prednisone and prednisolone present in serum were adjusted with respect to the deuterated internal standards and analyzed against their respective matrix-matched standard curve also adjusted against internal standard. Each feed sample was spiked at 4 concentrations so that a standard addition method could be used to calculate sample concentration. Retention times were: Prednisone, 0.95 min; Prednisone-*d*7,0.86 min; Prednisolone, 0.91min.

Extraction

For feed, 1 g of sample was combined with 3 mL acetonitrile and homogenized in a Precellys Evolution homogenizer (Bertin Technologies, Redondo Beach, CA) at 7500 RPM twice at 45 sec with a 30 sec

pause between. For serum, 200 µL of sample was combined with 600 µL acetonitrile, mixed by vortexing, and centrifuged to remove insoluble particulates. Feed homogenate supernatants and the serum:acetonitrile mixtures were passed through a 0.2 µm Pall Bio-inert filter (Pall Corporation, VWR International, Radnor, PA) by centrifugation in a Beckman Coulter Microfuge 16 (Beckman Coulter Life Sciences, Indianapolis, IN). From the eluate, 250 µL was transferred to an autosampler vial and submitted for chromatography.

Quantitative LC-MS/MS

Analyte separation was achieved using a Shimadzu LC30AD ultrahigh-performance liquid chromatography (UHPLC) system (Shimadzu, Kyoto Japan) followed immediately by a Phenomenex (Torrance, CA) Kinetex Biphenyl 100 angstrom column (30 × 2.1 mm i.d., 2.6 µm). Identification and quantitation were accomplished using an ABSciex 6500+ triple quadrupole mass spectrometer (ABSciex, Framingham, MA) with an electrospray ionization source in the negative ionization mode. Chromatographic separation and analyte detection settings are provided in Table 1. A formate adduct of Prednisone and Prednisolone generated by this procedure were used to establish MRM settings.

Table 1. Shimadzu LC30AD separation and ABSciex 6500+ Instrument Settings. MRM settings for Prednisone and Prednisolone were adopted from pre-established LC-MS/MS methodologies reported by Methlie *et al.,* 2013.

**Mobile phase**	**A: 0.1% formic acid in water**	**B: 0.1% formic acid in acetonitrile**
**Gradient (minutes)**	**Percentage**	**Percentage**
1.0	70	30
7.0	0	100
7.5	70	30
8.0	70	30

Injection Volume: 10 µL
Flow Rate: 0.5 mL/min

Molecular Mass<br>MRM Setting	Prednisone<br>358.43	Prednisone-*d*7<br>365.47	Prednisolone<br>360.44
Qualifier 1	*m/z* 403 -> 357		*m/z* 405 -> 295
Qualifier 2	*m/z* 403 -> 299	*m/z* 410 -> 110	*m/z* 405 -> 45
Quantifier	*m/z* 403 -> 327	*m/z* 410 -> 341	*m/z* 405 -> 329
LOD	0.5 ug/g		0.1 ng/mL
**LOQ**	5 ug/g		1 ng/mL

Reference:

Methlie, Paal, Steinar Hustad, Ralf Kellman, Bjørg Almås, Martina M. Erichsen, Eystein S. Husebye, and Kristian Løvås. "Multisteroid LC–MS/MS assay for glucocorticoids and androgens and its application in Addison's disease." *Endocrine connections* 2, no. 3 (2013): 125-136.

Supplementary Table 1. Experimental diet formulation

	Experimental Diet			
	CON (P0)	low PR (PL)	medium PR (PM)	high PR (PH)
Ingredient	(g/kg total diet)			
Corn Starch	398	398	398	398
Maltodextrin (dyetrose)	132	132	132	132
Sucrose	100	100	100	100
Cellulose	50	50	50	50
Casein	100	100	100	100
L-Cysteine	3	3	3	3
Corn Oil	10	10	10	10
High-Oleic Safflower Oil	60	60	60	60
AIN93G Mineral Mix	35	35	35	35
AIN93G Mineral Mix	10	10	10	10
Choline Bitartrate	3	3	3	3
TBHQ Antioxidant	0.01	0.01	0.01	0.01
	(mg/kg total diet)			
Prednsione	0	5	15	50

Supplementary Table 2. Criteria for defining moribund condition of silica-treated NZBWF1 mice[a]

Criteria	Method	Response	Score Assigned
Proteinuria[1,2]	Weekly monitoring	<2000 mg/dl = negative (NEG)	0
		>2000 mg/dl = positive (POS)	1
	Note: proteinuria scoring is cumulative over time		
	e.g., POS for proteinuria at 22 wks of age and POS at 23 wks of age = cumulative score of 2		
Weight Loss[2,3,4,5]	Weekly body weight	<10% loss = NEG	0
		10-14% loss = POS	2
		>15% loss = POS	4
	Note #1: loss of body weight will be calculated relative to body weight at onset of nephritis		
	Note #2: onset of nephritis defined as = 300 mg/dl proteinuria (determined in above measurements)		
Dyspenia[1,3,4,5,*]	Daily health check	Qualitative - NO	0
		Qualitative - YES	1
Ambulation[3,4,5,*]	Daily health check	Qualitative - NO	0
		Qualitative - YES	4
Rough Coat[3,4,5,*]	Daily health check	Qualitative - NO	0
		Qualitative - YES	1

[a] Table was reviewed by CAR Veterinarian Dr. Danielle Ferguson at Michigan State University. Mice receiving a score of 4 or more were euthanized. References for criteria previously published indicated by superscripts and noted below.

[1]Gardet et al. (2016) PLOS ONE. 11(10): e 0164423
[2]Frese-Schaper et al. (2010) J Immunology 184 (4) 2175-2182
[3]Toth, L. (2000). Journal of the Institute for Laboratory Animal Research (2000) 41 (2): 72-29
[4]Toth, L. (1997). Contemp. Top Lab Anim Sci 36, 44-48
[5]Tomasovic et al. (1988). Lab Animal 17:31-34,1998.
*The IACUC Handbook (2014) CRC Press. 3rd Edition

Supplementary Table 3. List of Kits, Reagents, and Chemicals

Reagent/Assay	Vendor	Catalog Number	Lot Number
Prednisone	Spectrum Chemical	PR103	4HF0028
Prednisolone	Toronto Research Chemicals		
Urine InstaTest Reagent Strips	Cortez Diagnostics	URS-2P	82721
AIN-93G Purified Rodent Diet without Vitamin Mix	Dyets Inc.	110700	
AIN-93VX Vitamin Mix	Dyets Inc.	310025	
LouAna Safflower Oil	LouAna Oils		
Mazola Corn Oil	Mazola		
Polyclonal Rabbit Anti-CD3 Antibody	Abcam	ab5690	
Monoclonal Rat Anti-Mouse CD45R Antibody	BD Pharmingen	550268	
Polyclonal Goat Anti-IgG Antibody	Bethyl Labs - FORTIS Life Sciences	A-90-100A	
Diff-Quick	Fisher Scientific		
XT_Mm_AIProfiling_CSO	NanoString	115000269	XT-CSO-MAIP1-12
nCounter Master Kit	NanoString	100054	NAA-AKIT-048

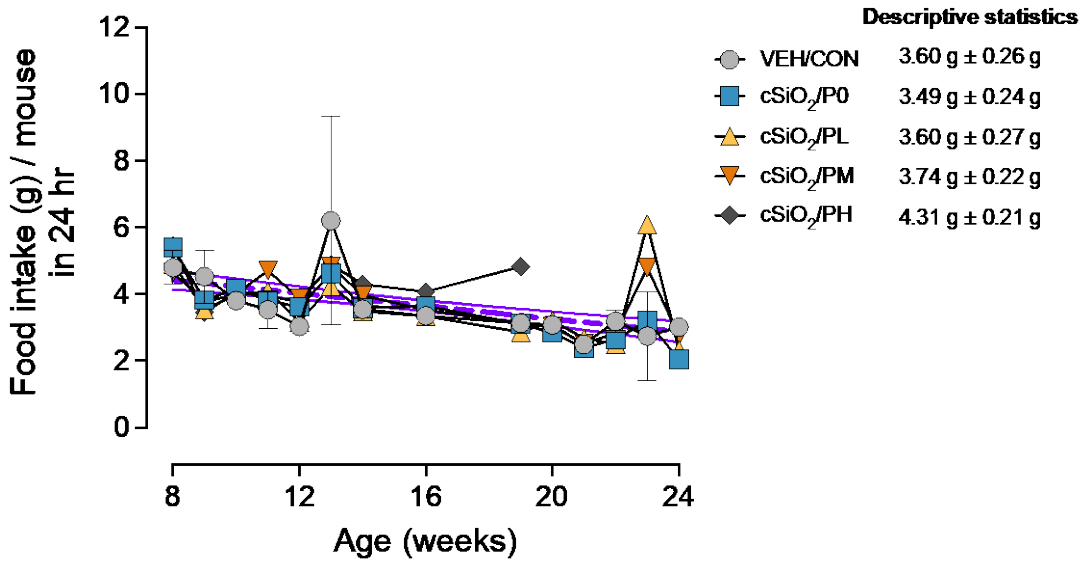

Supplementary Figure 1. Prednisone administration did not result in food refusal throughout the duration of the study. The amount of diet consumed between Monday morning and Tuesday morning (24 hr) was measured 14 out of 16 weeks prior to the Cohort A sacrifice date. Dietary intake per mouse for Cohort A was estimated by 1) calculating the difference between total food administered per cage Monday and total food remaining 24 hr later, then 2) dividing the difference by the number of mice per cage (n=4). Data points represent the estimated diet eaten per mouse for two separate cages (n=2) at each time point. Descriptive statistics represent the 14-week average dietary intake per mouse (g)/24 hr ± SEM for the VEH/P0, cSiO_2/P0, cSiO_2/PL, and cSiO_2/PM groups, and the 9-week average ± SEM for the cSiO_2/PH group. Significant differences were not detected between individual slopes for each treatment group based on nonlinear regression analysis. The combined slope of -0.09113 is shown with shaded bands around the regression line illustrating 95% confidence intervals.

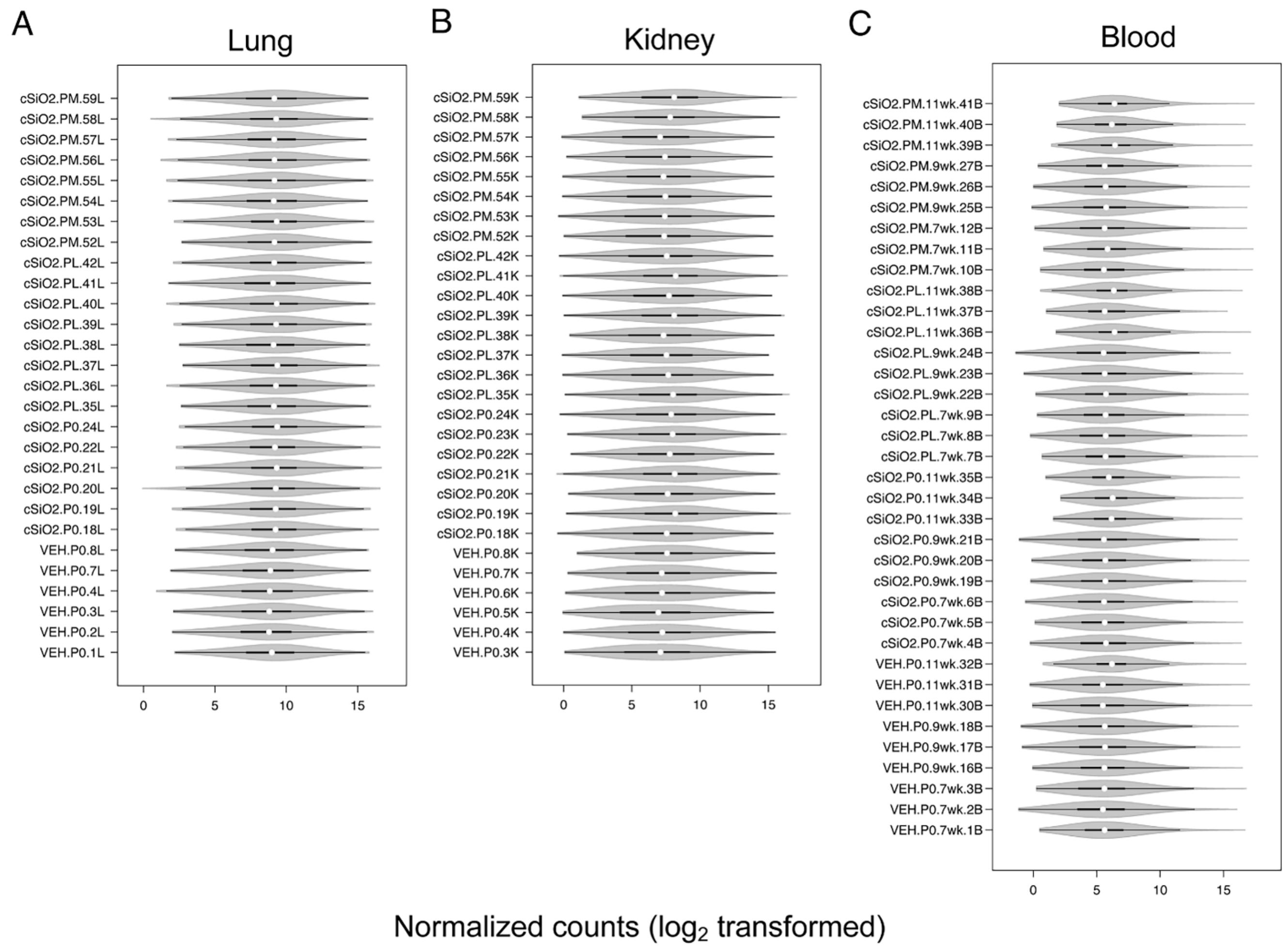

Supplementary Figure 2. Violin plots depicting distribution of normalized counts obtained using NanoString Autoimmune Profiling gene panel for lung **(A)** and kidney **(B)** tissues obtained 14 weeks post $cSiO_2$ instillation and **(C)** for whole blood obtained 7-, 9-, or 11-weeks post $cSiO_2$ instillation. Within the violin distribution polygon, bars represent the 25th to 27th percentile and the white circle indicates the median. Values are shown as the log_2 transformation of normalized counts. Values <20 counts (log_2=4.32) were excluded from analyses.

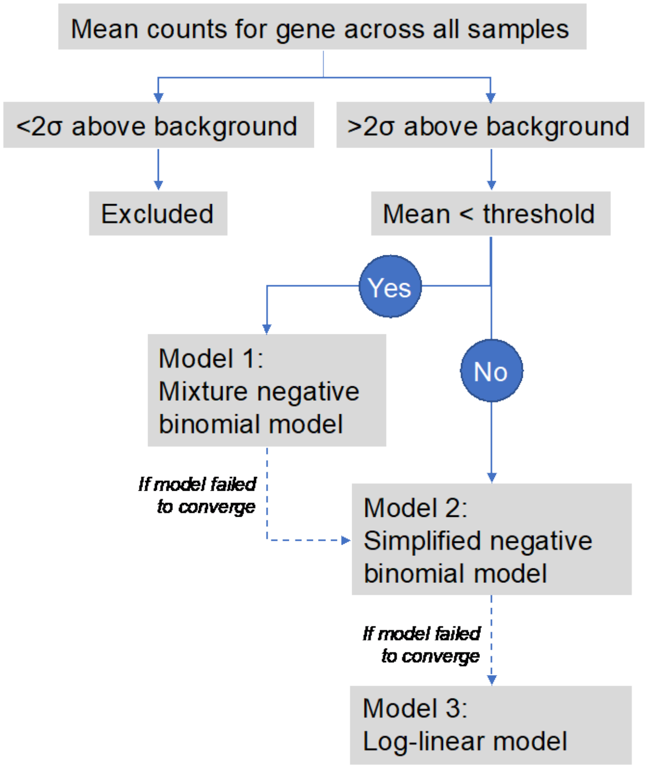

Supplementary Figure 3. Process flow for differential gene expression analysis using nSolver. As outlined by the supplied user manual[1], the mean of the gene across all samples was compared against the threshold, which is set as 10 times the background signal. If the gene mean was below the threshold, the mixture negative binomial (*MLE* function in R, Wald test to calculate a p value) was applied; if model 1 did not converge, the simplified model in 2 (*glm.nb* function in R/Mass) was applied instead. If the gene mean was above the threshold, the mixture model in 1 was simplified to model 2. If model 2 did not converge, the log-linear model 3 was used (*lm* function in R).

[1] NanoString Technologies, Inc. (2018) nCounter Advanced Analysis 2.0 Plugin for nSolver Software User Manual, vers. Jan 2018 (MAN-10030-03). Accessed at www.nanostring.com

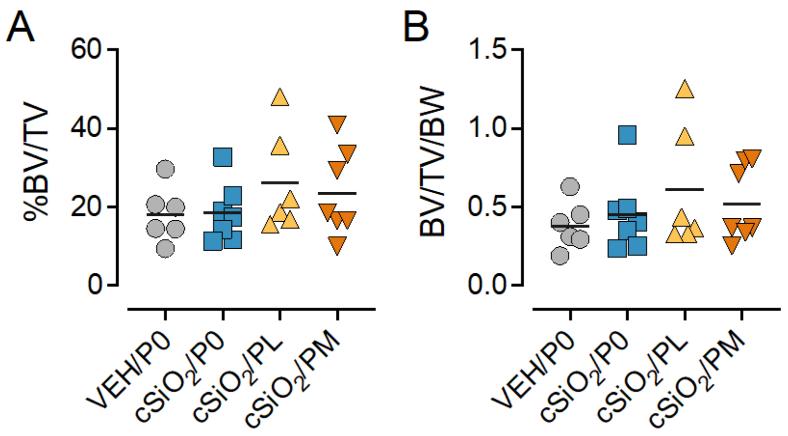

Supplementary Figure 4. Prednisone does not significantly reduce bone density in $cSiO_2$-exposed NZBWF1 mice. Dietary administration of prednisone did not induce trabecular bone loss. Femurs collected from mice at time of necropsy (25 wks of age) were analyzed using µCT. Box-plot whiskers represent the minimum and maximum levels of bone density. No significant differences were detected between VEH/P0 and $cSiO_2$/P0 groups, or between $cSiO_2$/P0 and prednisone-fed groups. BV/TV = bone volume/total volume; BV/TV/BW = bone volume/total volume/body weight.

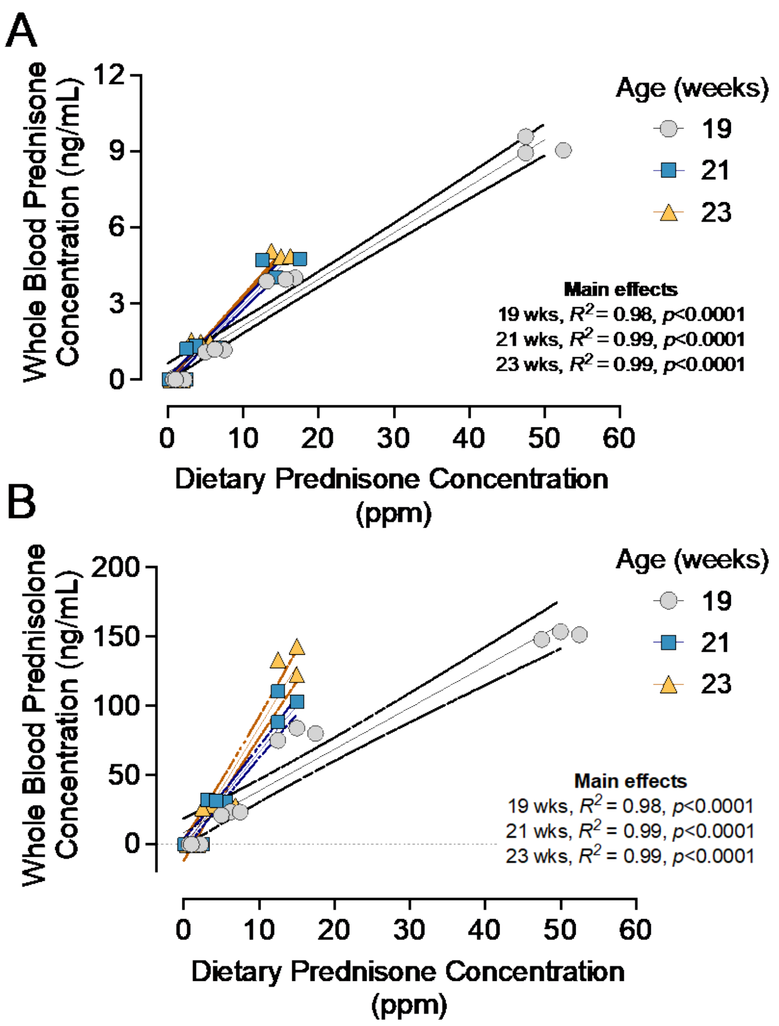

Supplementary Figure 5. Prednisone concentration detected in whole blood is predictable based on dietary prednisone concentrations at multiple timepoints. (A) Levels of prednisone and its active metabolite, prednisolone **(B)**, were detected in whole blood samples taken from mice 19, 21, 23 wk of age determined via LC-MS/MS. Samples were pooled within each cage (n=3/cage), with 3 cages per treatment group resulting in an n=3/group for analysis. For regression analyses, R^2 and p-values are reported for each timepoint. Shaded bands around regression lines represents 95% confidence intervals.

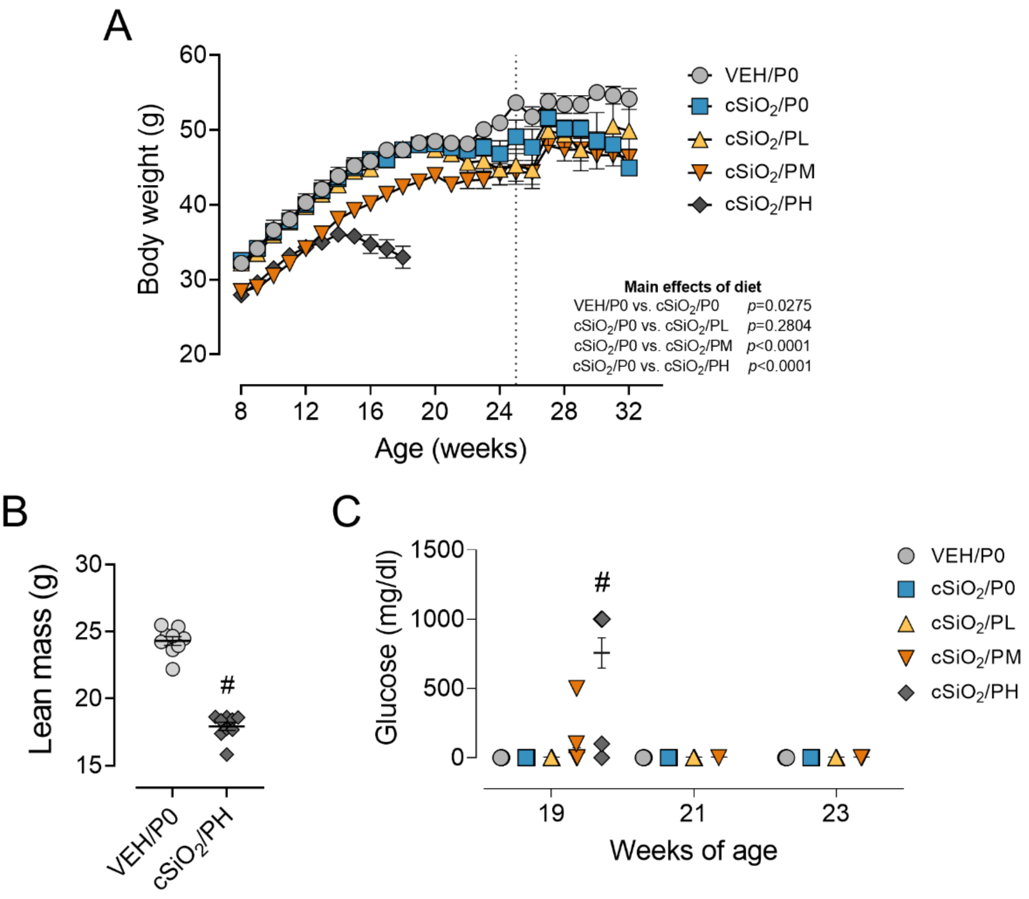

Supplementary Figure 6. High dose prednisone treatment (PH) caused secondary toxicity in $cSiO_2$-instilled mice. (A) Mice from Cohort A and Cohort B were weighed weekly starting at 8 wk of age. Values are shown as mean ± SEM. Unlike PL and PM groups, mice in the PH group experienced weight loss starting at approximately 15-16 wk of age. Body weights were significantly lower in mice provided the PM and PH diets up to 25 wk of age (at time of first necropsy, dashed line) or 18 wk, respectively, as compared to the $cSiO_2$/P0 group. **(B)** At 17 wk of age (5 wk PI) high dose prednisone treatment resulted in significant lean muscle loss compared to VEH/P0 mice. [#]Indicates $p<0.05$ as determined using a two-tailed Student's t-test. **(C)** At 19 wk of age (7 wk PI) mice were evaluated for urine glucose using clinical dipsticks. High dose prednisone treatment resulted in elevated urine glucose levels compared to all other groups. [#]Indicates $p<0.05$ compared to $cSiO_2$/P0 as determined by Kruskal-Wallis nonparametric test.

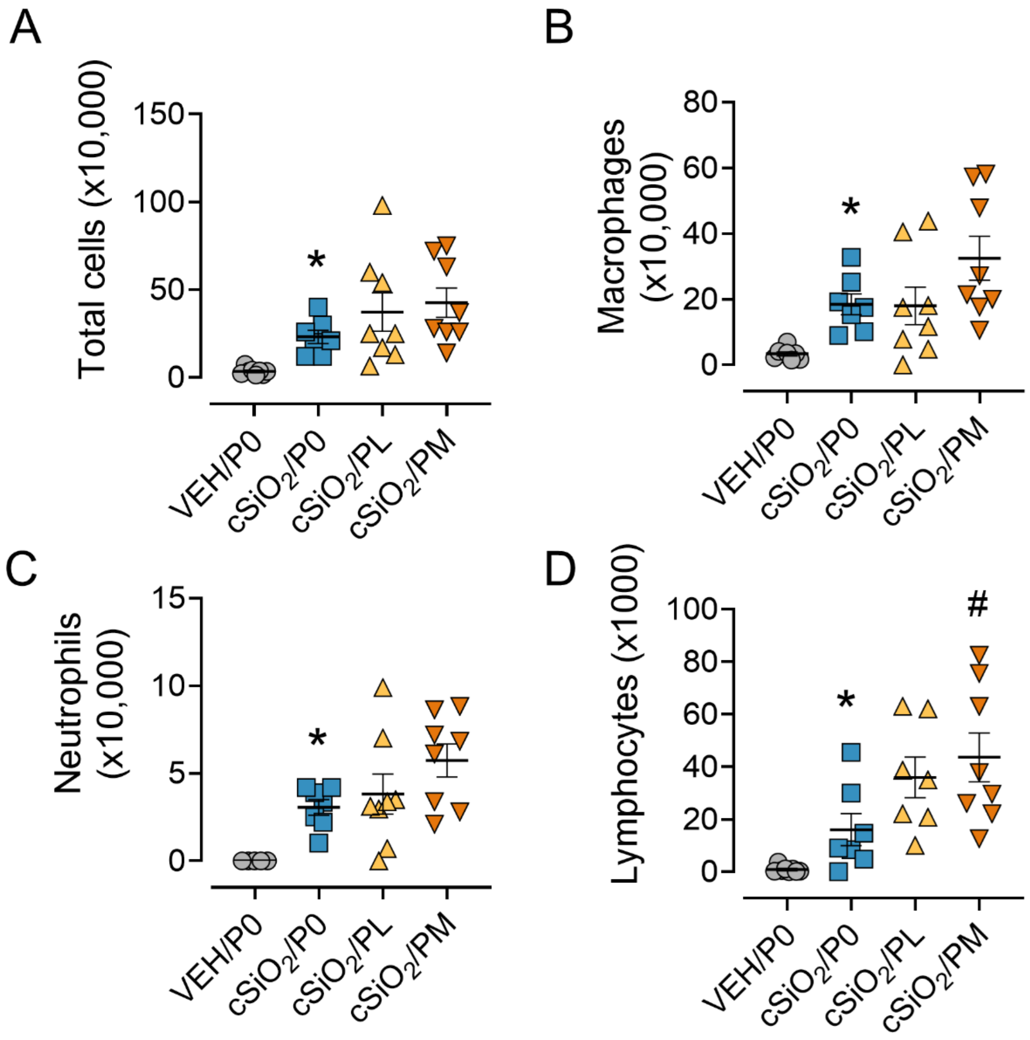

Supplementary Figure 7. Low and medium dose Prednisone did not reduce cSiO_2-triggered BALF inflammatory cell counts. SiO_2 instillation caused significant increases in **(A)** total cells, **(B)** macrophages, **(C)** neutrophils, and **(D)** lymphocytes cellular inflammation in the BALF, but these responses were unaffected by PL or PM diets. *Indicates $p<0.05$ for VEH/P0 vs cSiO_2/P0; #indicates $p<0.05$ for cSiO_2/P0 vs cSiO_2/PL or cSiO_2/PM group.

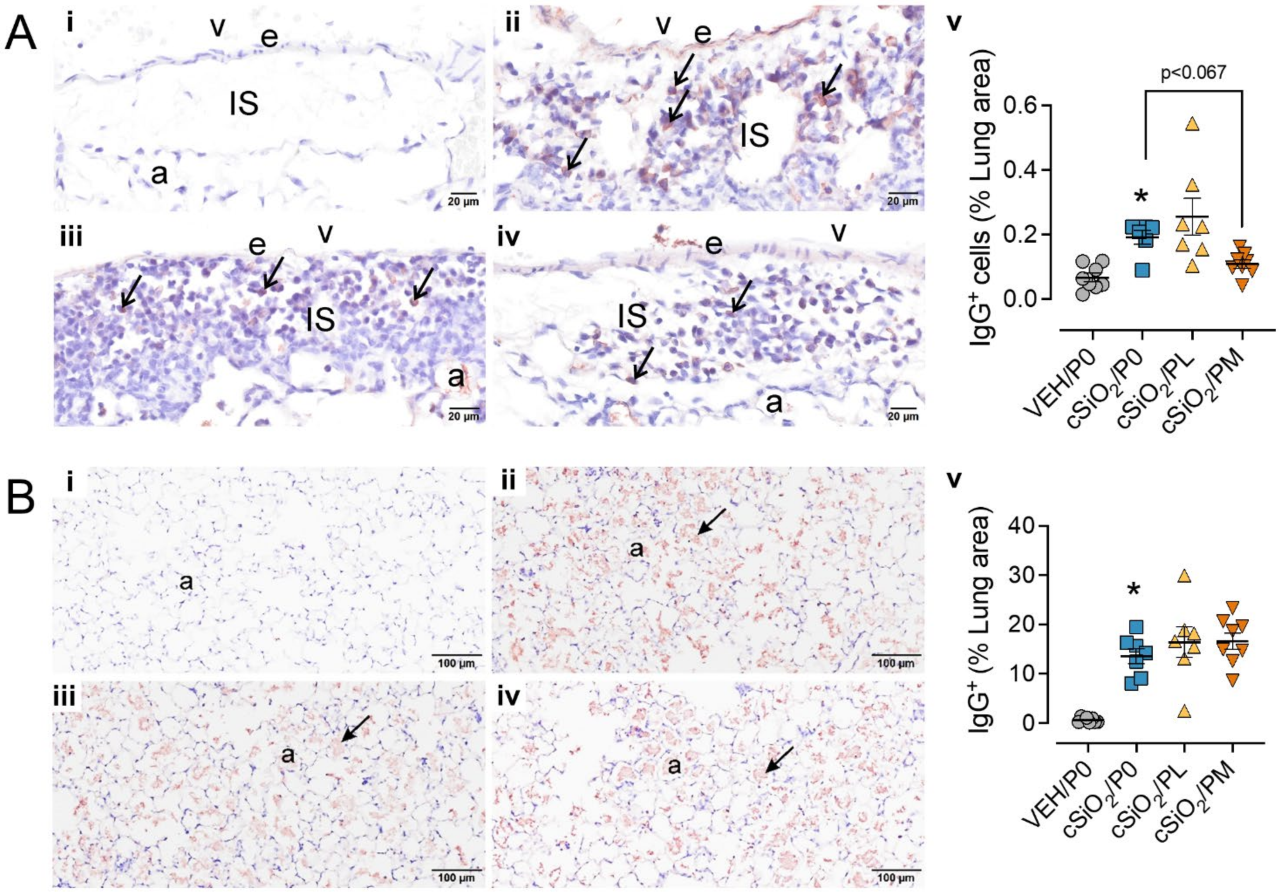

Supplementary Figure 8. Prednisone reduces pulmonary IgG+ plasma cells but not overall IgG deposition. Light photomicrographs of perivenous ectopic lymphoid structures (ELS) immunohistochemically stained for **(A)** IgG+ B plasma cells (arrows) and **(B)** alveolar parenchyma immunohistochemically stained for extracellular IgG+ proteinaceous material (arrows) in alveolar airspace (a) in (i) VEH/P0, (ii) $cSiO_2$/P0, (iii) $cSiO_2$/PL, and (iv) $cSiO_2$/PM mice. e, endothelium; v, venous lumen; IS, perivascular interstitial space; a, alveolus. Graphical representation of morphometrically determined lung density of IgG+ plasma cells (A.v) and total IgG (B.v). PM treatment modestly reduces pulmonary IgG+ plasma cell density (A.v) but has no effect on extracellular IgG (B.v). Tissues counterstained with hematoxylin. *Indicates $p<0.05$ for VEH/P0 vs $cSiO_2$/P0; $^{\#}$indicates $p<0.05$ for $cSiO_2$/P0 vs $cSiO_2$/PL or $cSiO_2$/PM group.

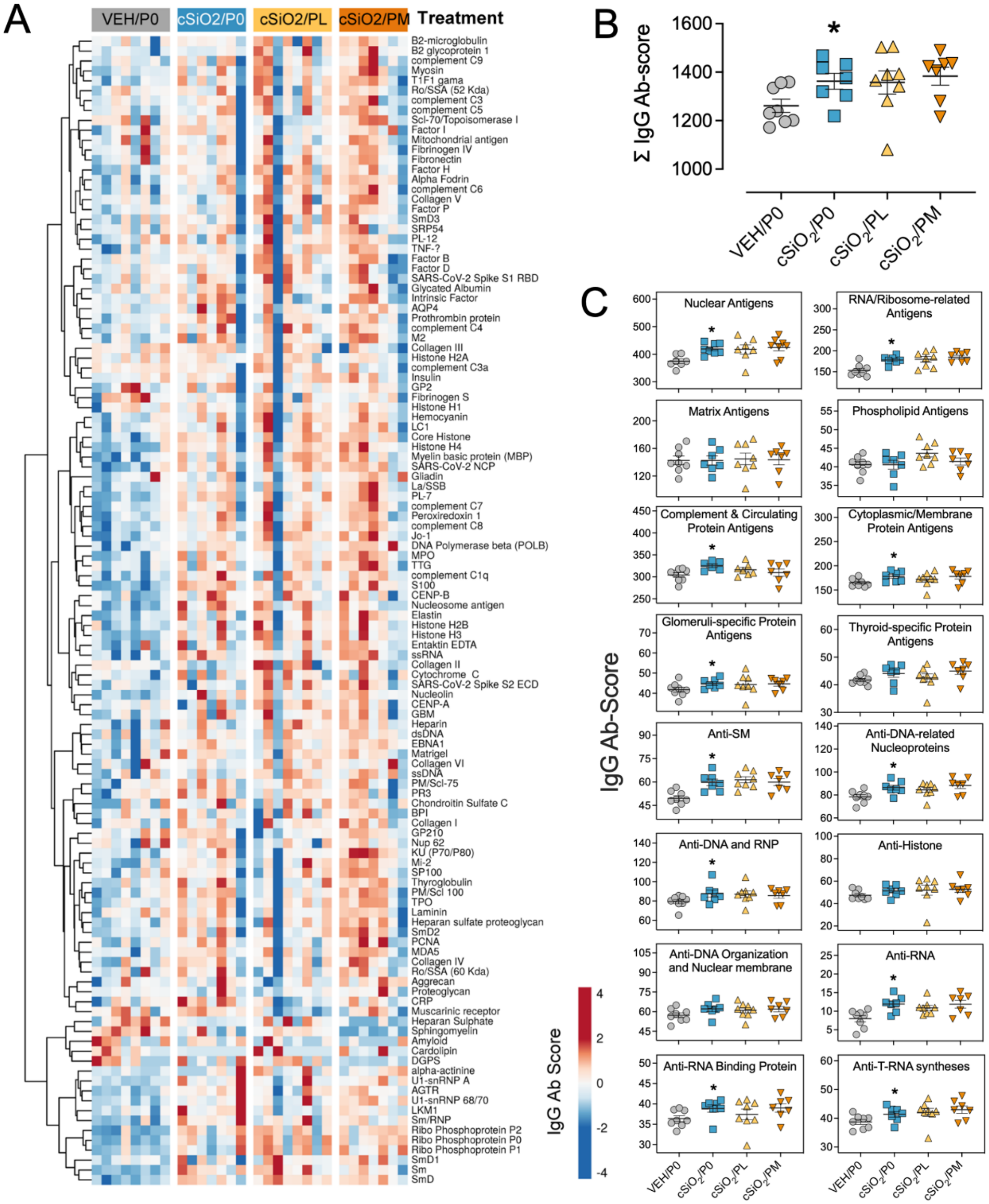

Supplemental Figure 9. Prednisone treatment is not effective in reducing $cSiO_2$-induced AAbs in the plasma. AAbs production was measured in plasma using Cohort A samples collected at time of necropsy (14 wk pi). **(A)** Heat map illustrates unsupervised clustering (Euclidian distance method) of 122 AAbs depict Ab-score values for IgG expression in plasma. Scale bar values reflect the range of variance-stabilized Ab scores, which were centered across rows. **(B)** Prednisone did not reduce overall IgG levels in the plasma compared to $cSiO_2$ controls. **(C)** Prednisone did not alter reduce certain classes of AAbs in the plasma compared to the $cSiO_2$/P0 positive control group. * $p<0.05$ for $cSiO_2$/P0 compared to VEH/P0; $^{\#}$ $p<0.05$ for $cSiO_2$/PL or $cSiO_2$/PM compared to $cSiO_2$/P0 as described in Materials and Methods.

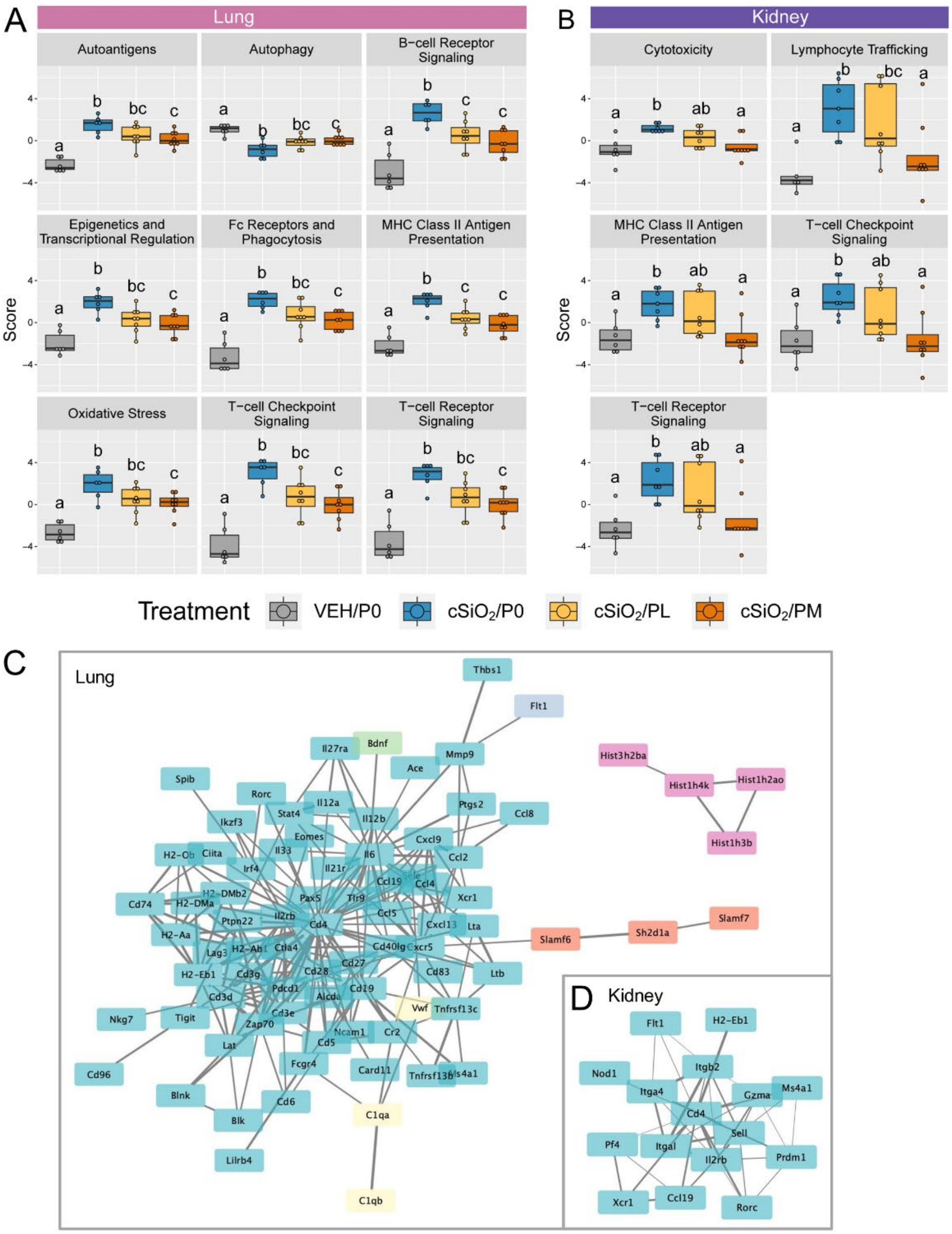

Supplementary Figure 10. Effect of prednisone treatment on select autoimmune pathways in lung and kidney tissues of mice 14 weeks post instillation with $cSiO_2$. (A-B) Pathway Z scores are presented as Tukey box-plots for select pathways of interest for lung **(A)** or kidney **(B)** tissues. Within a tissue type, different letters indicate that the treatment groups are significantly different ($p<0.05$) as described in Materials and Methods. **(C-D)** Network visualizations of genes significantly affected by prednisone treatment in lung **(C)** or kidney **(D)** tissues. Network interactions for genes differentially regulated by medium-dose prednisone treatment were modeled using the STRING database (string-db.org) with a minimum required interaction score >0.7, and clusters were identified using the Markov Cluster (MCL) algorithm with inflation parameter of 1.5. Networks were visualized in Cytoscape, and edge widths reflect the combined interaction score (thicker edge indicates higher score). Abbreviations: $cSiO_2$, crystalline silica; P0, zero prednisone; PL, low-dose prednisone; PM, medium-dose prednisone; VEH, vehicle control.

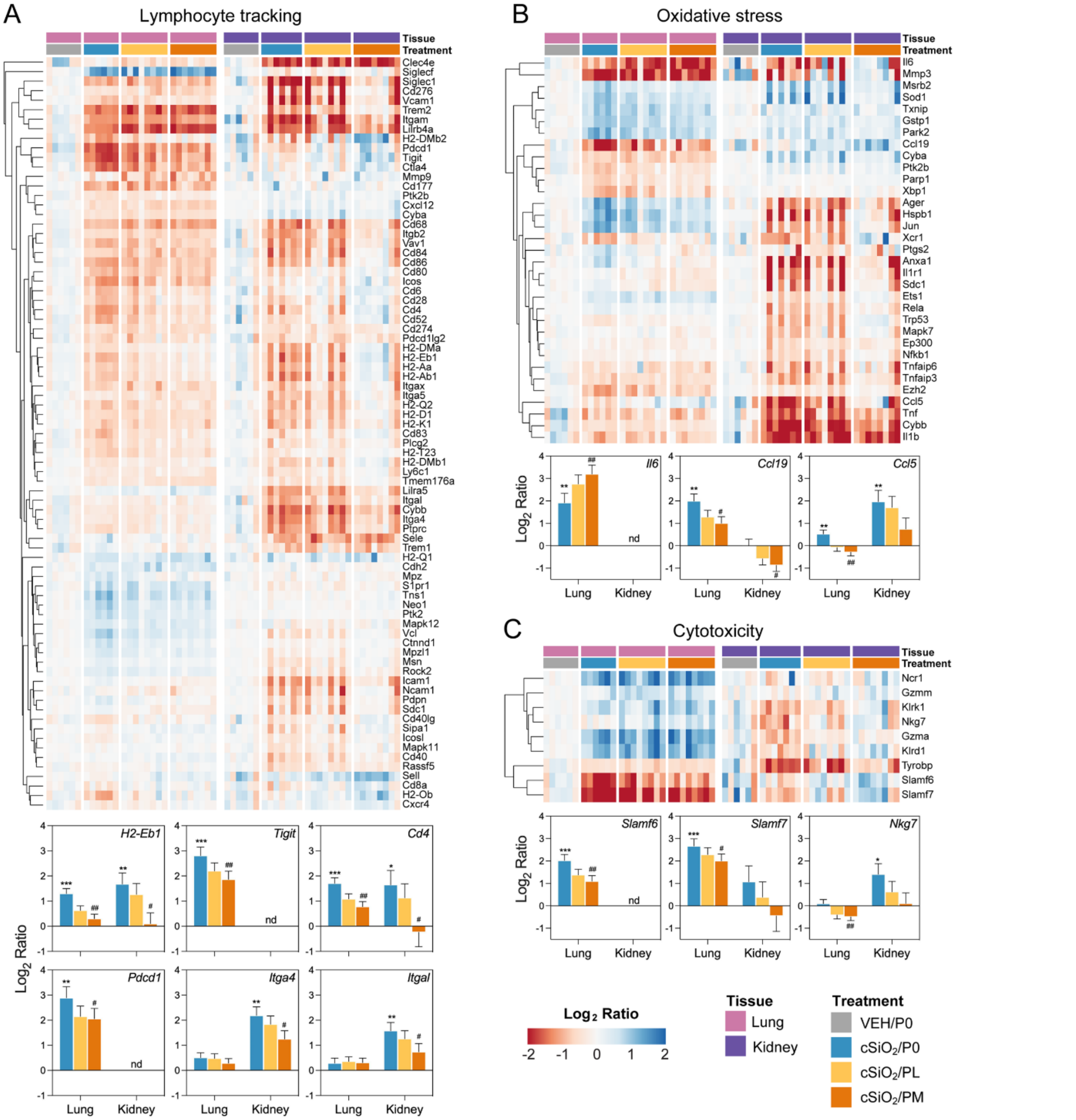

Supplementary Figure 11. Comparison of prednisone-responsive genes associated with (A) lymphocyte tracking, (B) oxidative stress, and (C) cytotoxicity pathways in lung or kidney tissues 14 weeks post instillation with $cSiO_2$. Gene expression data were obtained using the NanoString Autoimmune Profiling gene panel and are shown as log_2 ratios for $cSiO_2$/P0, $cSiO_2$/PL, and $cSiO_2$/PM treatment groups with respect to the tissue-matched VEH/P0 control group (log_2 ratio = 0). For each pathway, heatmaps with unsupervised hierarchical clustering (Euclidian distance method) by gene show log_2 expression values for all genes identified as differentially expressed in response to either $cSiO_2$ exposure or medium-dose prednisone (FDR q<0.05, 1.5-fold change) in either of the selected tissues. The mean log_2 ratio values + SEM for selected genes of interest are also shown for each pathway. For $cSiO_2$/P0 as compared to VEH/P0, *, FDR-corrected q<0.05; **, q<0.01; and ***, q<0.001. For $cSiO_2$/PL or $cSiO_2$/PM vs $cSiO_2$/P0, #, FDR-corrected q<0.05; ##, q<0.01; and ###, q<0.001. See Supplementary File 1 for test specifications and FDR-corrected q-values for all genes in the panel for all comparisons. Abbreviations: $cSiO_2$, crystalline silica; P0, zero prednisone; PL, low-dose prednisone; PM, medium-dose prednisone; VEH, vehicle control.

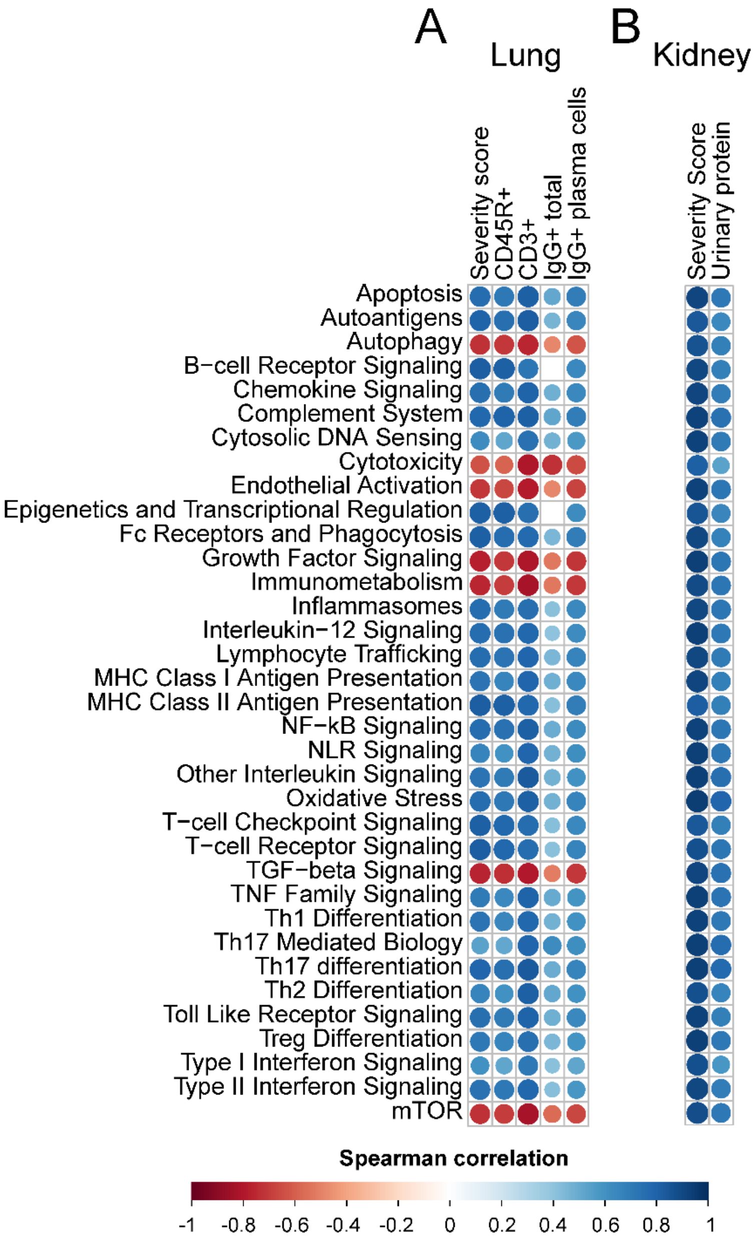

Supplemental Figure 12. Correlation analyses of all pathways represented in the NanoString autoimmune profiling panel. For all treatment groups, spearman ρ values were calculated by correlating pathway Z scores with **(A)** lung severity score or the percent positive staining for CD45R+, CD3+, or IgG+ in lung tissue or IgG+ in lung tissue plasma cells; or **(B)** kidney severity score, kidney blood urea nitrogen (BUN), or kidney urinary protein. Significant correlation values ($p<0.05$) are represented as circles colored by the correlation value (blue, positive; red, negative); non-significant correlations are indicated by blank cells.

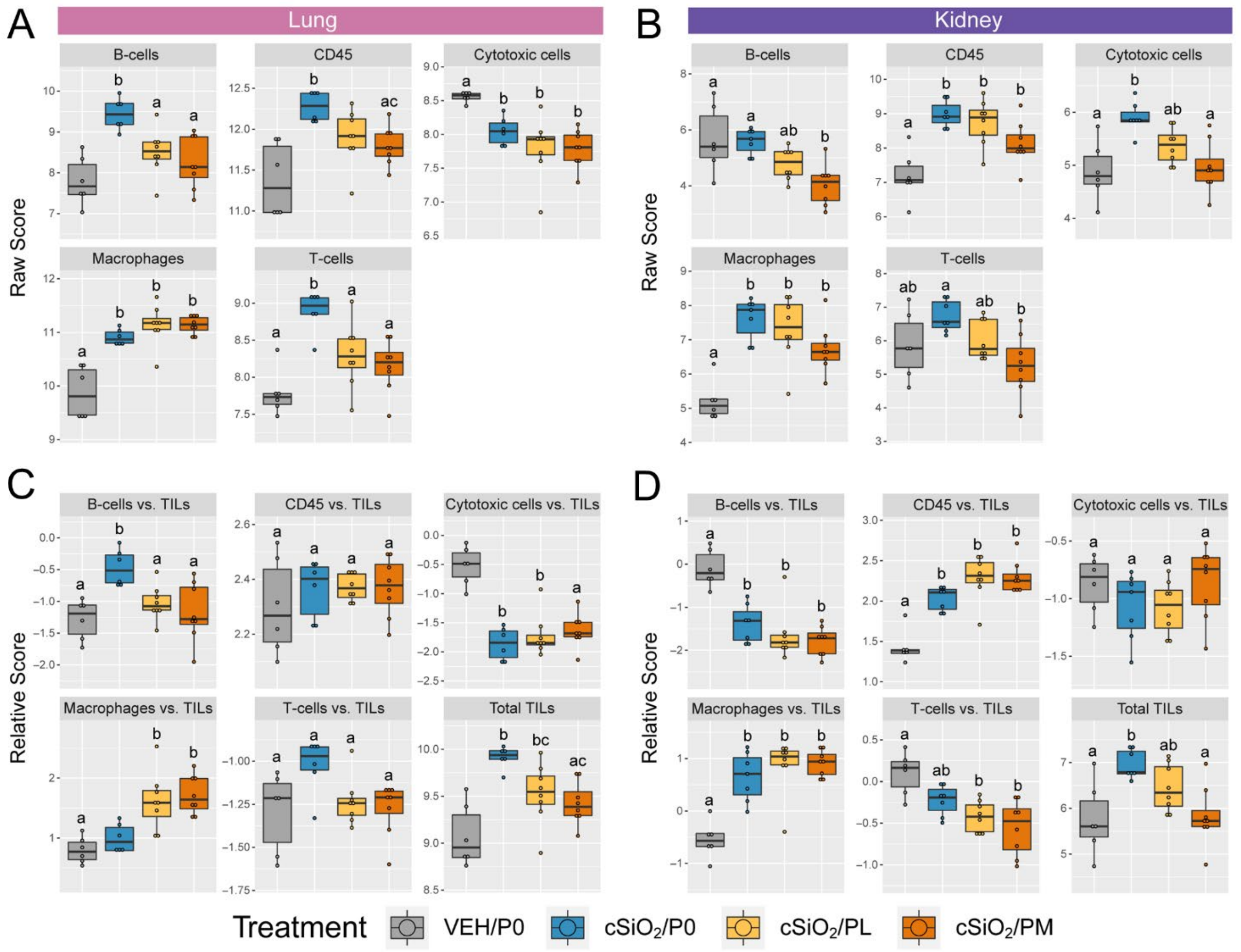

Supplementary Figure 13. Profiling of immune cell types in lung or kidney tissues from mice 14 weeks post instillation with $cSiO_2$. Data shown are the log_2 raw scores **(A-B)** or relative scores with respect to total infiltrating leukocytes (TILs) **(C-D)** for the indicated cell types for lung (A,C) and kidney (B,D). Only those cell types passing quality control testing for correlation of marker gene expression for either tissue ($p<0.05$) are shown. Within a tissue type, different letters indicate that the treatment groups are significantly different ($p<0.05$) as described in Materials and Methods. Scores may be compared by treatment within a cell type, but comparisons across cell types are not appropriate for this method of quantitation.

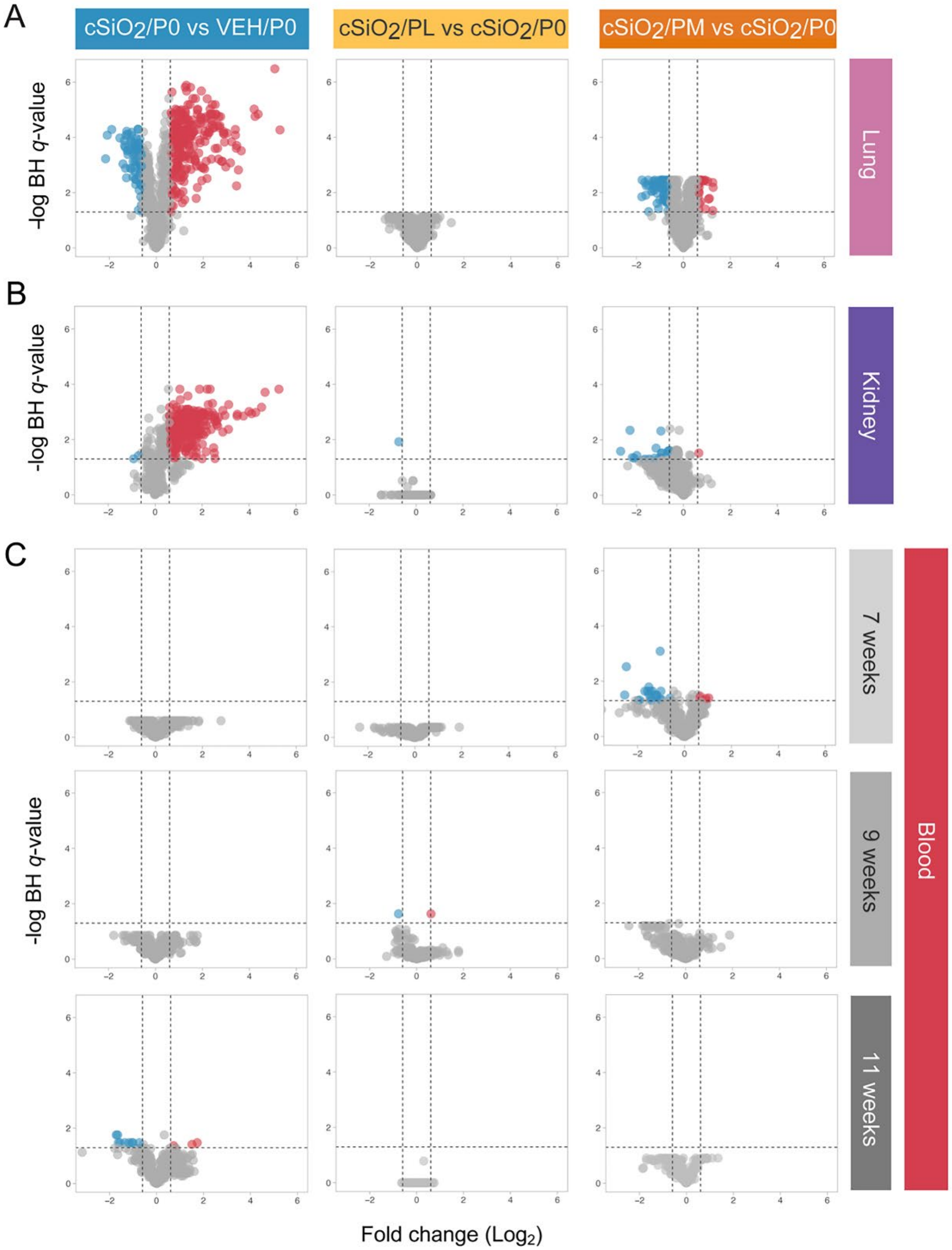

Supplementary Figure 14. Volcano plots depicting gene expression determined using the NanoString Autoimmune Profiling gene panel for **(A)** lung and **(B)** kidney tissues obtained 13 weeks post instillation with $cSiO_2$ or whole blood samples obtained 7-, 9-, or 11-weeks post instillation. Values shown are the fold change (log_2 ratio) for $cSiO_2$/P0 treatment vs tissue-matched VEH/P0 control group, or for low-dose ($cSiO_2$/PL) or medium-dose ($cSiO_2$/P0) prednisone versus tissue matched zero prednisone control ($cSiO_2$/P0) plotted against the $-log_{10}$ Benjamini-Hochberg FDR-corrected q-value. A significant difference in gene expression was inferred with 1.5-fold change (log_2 <-0.585 or >0.585) with $q<0.05$.

A

Timepoint & comparison	DEGs Up	DEGs Down
Week 7		
$cSiO_2$/P0 vs. VEH/P0	0	0
$cSiO_2$/PL vs $cSiO_2$/P0	0	0
$cSiO_2$/PM vs $cSiO_2$/P0	5	20
$cSiO_2$/PM vs $cSiO_2$/PL	0	0
Week 9		
$cSiO_2$/P0 vs. VEH/P0	0	0
$cSiO_2$/PL vs $cSiO_2$/P0	1	1
$cSiO_2$/PM vs $cSiO_2$/P0	0	0
$cSiO_2$/PM vs $cSiO_2$/PL	0	0
Week 11		
$cSiO_2$/P0 vs. VEH/P0	3	10
$cSiO_2$/PL vs $cSiO_2$/P0	0	0
$cSiO_2$/PM vs $cSiO_2$/P0	0	0
$cSiO_2$/PM vs $cSiO_2$/PL	0	0

B

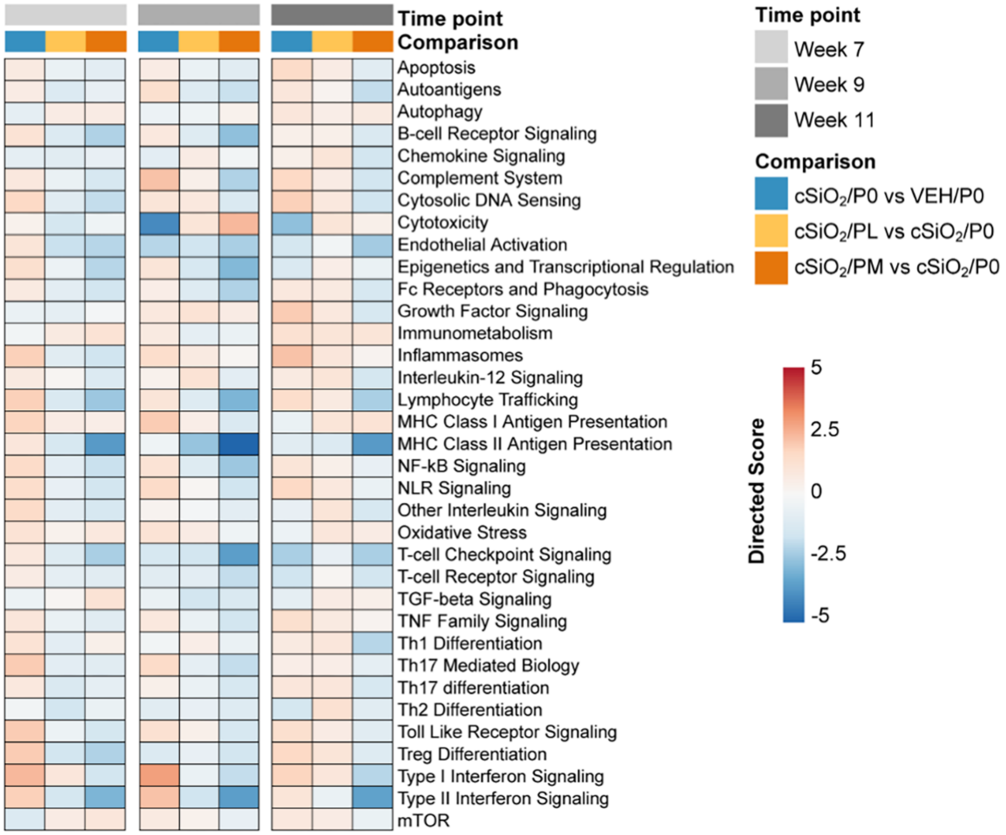

Supplementary Figure 15. Effect of prednisone treatment on $cSiO_2$-induced transcriptional changes in whole blood 7-, 9-, and 11-weeks post instillation. **(A)** Table indicating the number of differentially expressed genes (FDR *q*<0.05, 1.5-fold change) at each time point for each treatment comparison of interest. Venn diagrams and principal components analyses of whole blood sample data were not performed due to the low number of differentially expressed genes identified in blood samples. **(B)** Directed significance scores for select autoimmune pathways were determined using nSolver (see Materials and Methods) by comparing $cSiO_2$/P0 to timepoint-matched VEH/P0 control group or by comparing $cSiO_2$/PL or $cSiO_2$/PM treatments to timepoint-matched $cSiO_2$/P0 treatment group.

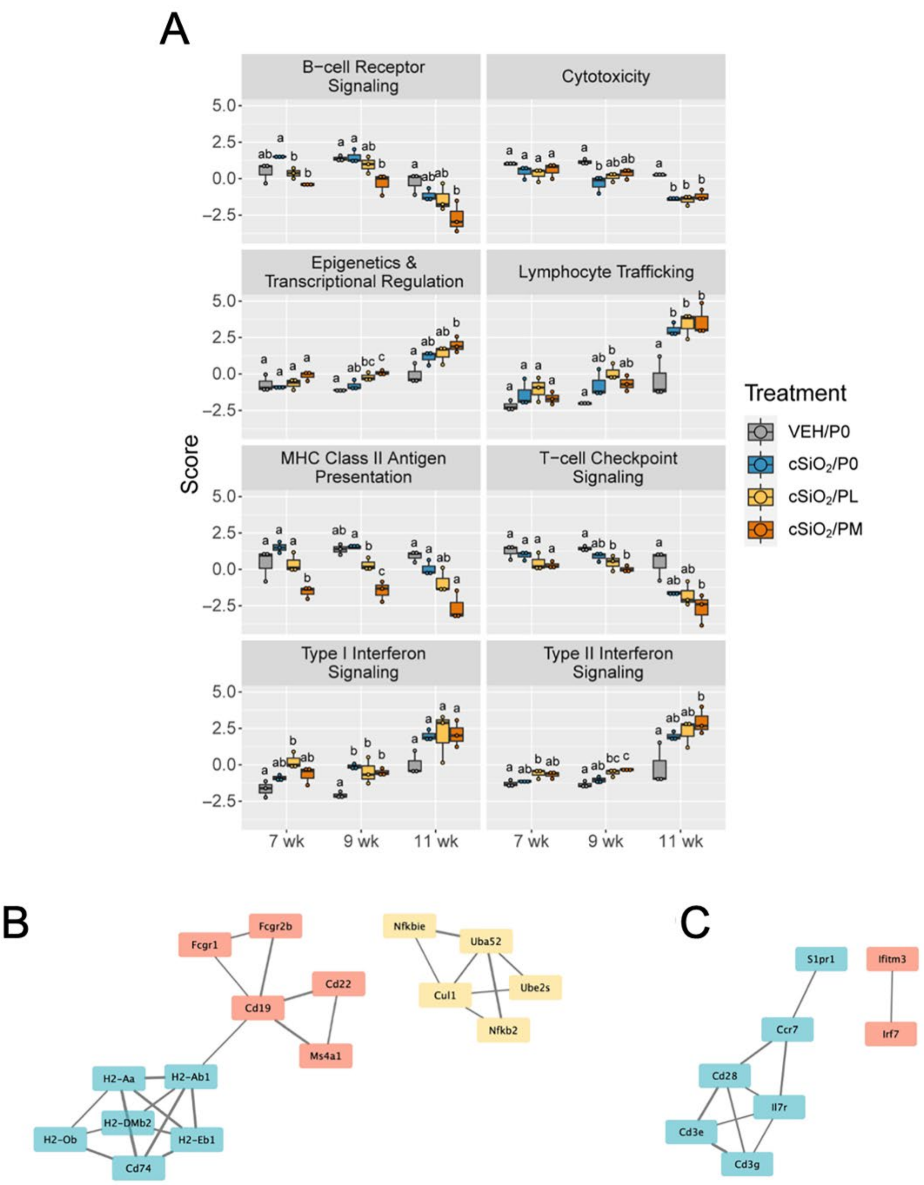

Supplementary Figure 16. Effect of prednisone treatment on select autoimmune pathways in whole blood obtained 7-, 9-, and 11-weeks post instillation with $cSiO_2$. **(A)** Pathway Z scores ae presented as Tukey box-plots (n=3) for select pathways of interest. Within each time point, different letters indicate that the treatment groups are significantly different ($p<0.05$) as determined by one-way ANOVA with Tukey HSD post-hoc test for multiple comparisons. **(B)** Network visualization of genes significantly affected by medium-dose prednisone compared to control (cSiO2/PM vs cSiO2/P0) in blood at 7-weeks post instillation. **(C)** Network visualization of genes significantly affected by $cSiO_2$ exposure ($cSiO_2$/P0 vs VEH/P0) at 11-weeks post-instillation. Networks were not considered for other comparisons or time points due to few or no identified differentially expressed genes. Network interactions were modeled using the STRING database (string-db.org) with a minimum required interaction score >0.7, and clusters were identified using the Markov Cluster (MCL) algorithm with inflation parameter of 1.5. Networks were visualized in Cytoscape, and edge widths reflect the combined interaction score (thicker edge indicates higher score).

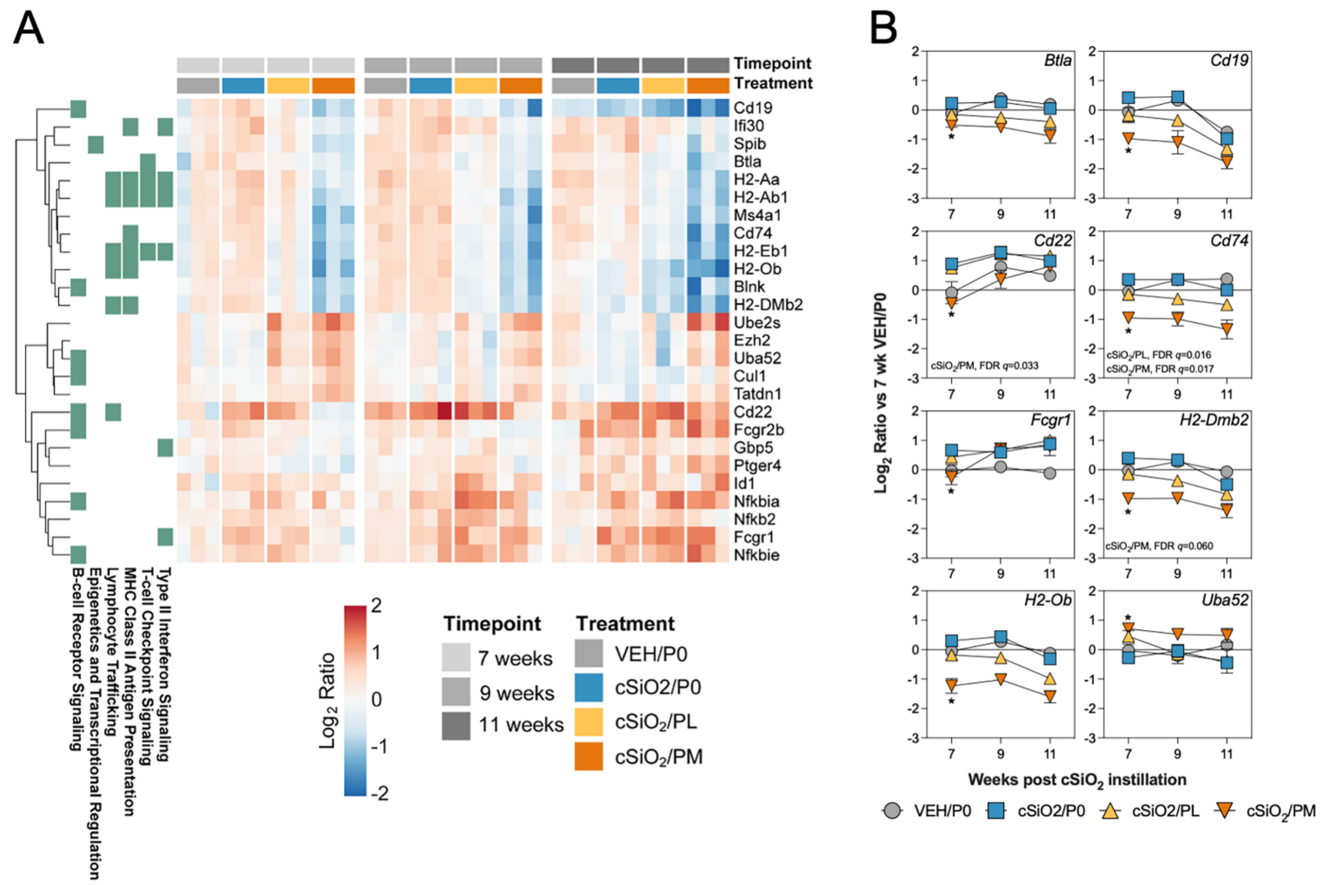

Supplementary Figure 17. Comparison of prednisone-responsive genes associated with selected pathways of interest in whole blood obtained 7-, 9-, and 11-weeks post instillation with $cSiO_2$. Gene expression data were obtained using the NanoString Autoimmune Profiling gene panel and are shown as log_2 ratios for $cSiO_2$/P0, $cSiO_2$/PL, and $cSiO_2$/PM treatment groups with respect to the 7-week, VEH/P0 control group (log_2 ratio = 0). **(A)** Heatmap with unsupervised hierarchical clustering (Euclidian distance method) by gene shows log_2 expression values for all genes identified as differentially expressed in response medium-dose prednisone (FDR q<0.05, 1.5-fold change) at any time point. Membership in autoimmune pathways is indicated to the left (green bar). **(B)** The mean log_2 ratio values + SEM for selected genes of interest are also shown. *Indicates FDR-corrected q<0.05 for $cSiO_2$/PM vs $cSiO_2$/P0. (No significant differences were observed for $cSiO_2$/P0 vs. VEH/P0 or $cSiO_2$/PL vs. cSiO2/P0 comparisons for these genes.) Main effects of the $cSiO_2$/PL or $cSiO_2$/PM treatments vs. $cSiO_2$/P0 are also indicated, when significant (or trending). See Supplementary File 1 for test specifications and FDR-corrected q-values for all genes in the panel for all comparisons.

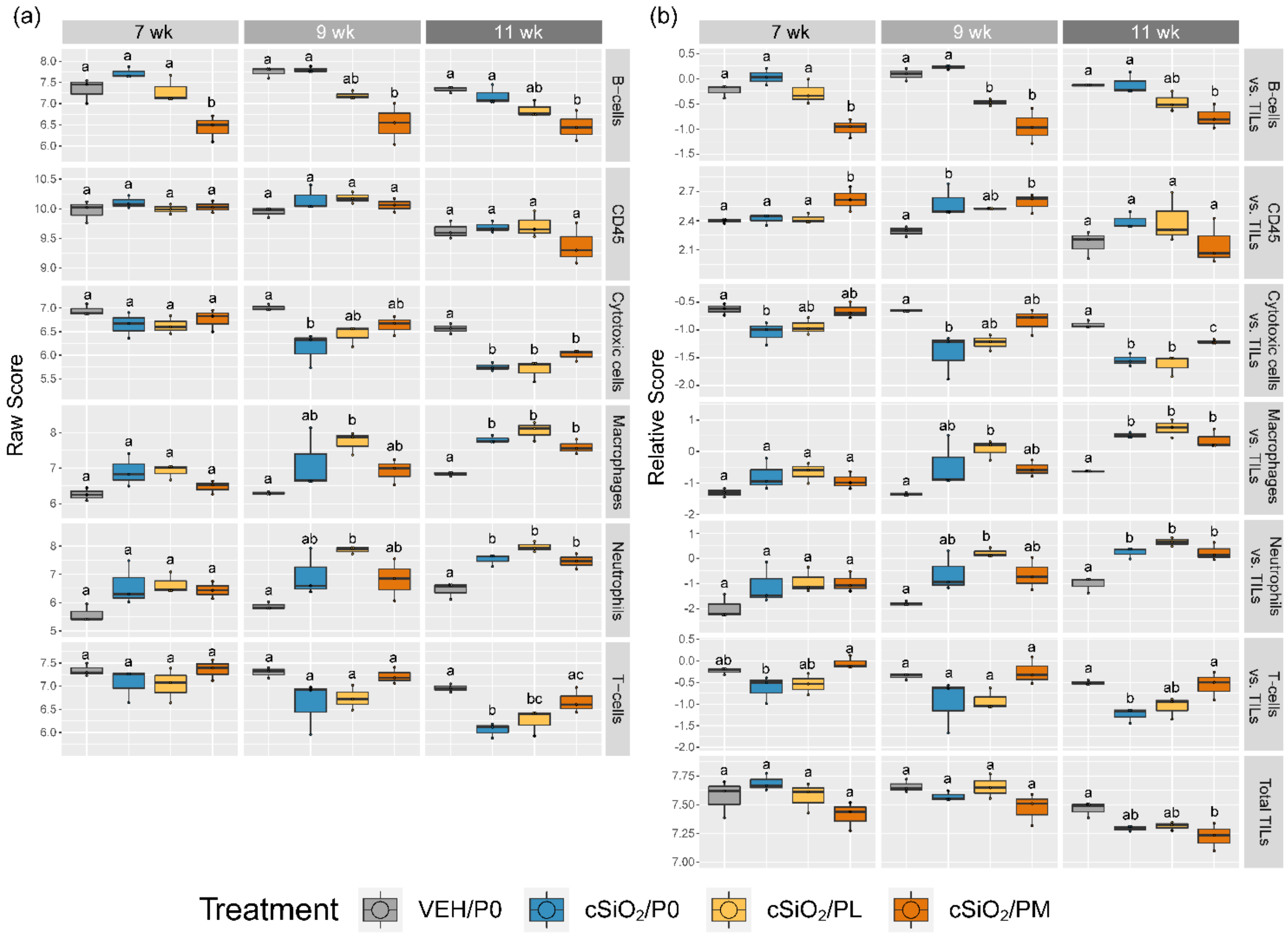

Supplemental Figure 18. Profiling of immune cell types in whole blood from mice 7-, 9-, or 11-weeks post instillation with $cSiO_2$. Data shown are the log_2 raw scores **(A)** or relative scores with respect to total infiltrating leukocytes (TILs) **(B)** for the indicated cell types. Only those cell types passing quality control testing for correlation of marker gene expression for either tissue ($p<0.05$) are shown. Within a tissue type, different letters indicate that the treatment groups are significantly different ($p<0.05$) as described in Materials and Methods. Scores may be compared by treatment within a cell type, but comparisons across cell types are not appropriate for this method of quantitation.
